# Prediction of anti-CD25 and 5-FU treatments efficacy for pancreatic cancer using a mathematical model

**DOI:** 10.1186/s12885-021-08770-z

**Published:** 2021-11-15

**Authors:** Sajad Shafiekhani, Hojat Dehghanbanadaki, Azam Sadat Fatemi, Sara Rahbar, Jamshid Hadjati, Amir Homayoun Jafari

**Affiliations:** 1 Departments of Biomedical Engineering, School of Medicine, Tehran University of Medical Sciences, Tehran, Iran; 2 Research Center for Biomedical Technologies and Robotics, Tehran, Iran; 3 Students’ Scientific Research Center, Tehran University of Medical Sciences, Tehran, Iran; 4 Metabolic Disorders Research Center, Endocrinology and Metabolism Molecular-Cellular Sciences Institute, Tehran University of Medical Sciences, Tehran, Iran; 5 Departments of Medical Immunology, School of Medicine, Tehran University of Medical Sciences, Tehran, Iran

**Keywords:** 5-FU, Anti-CD25, GUI, Fuzzy, ODE

## Abstract

**Background:**

Pancreatic ductal adenocarcinoma (PDAC) is a highly lethal disease with rising incidence and with 5-years overall survival of less than 8%. PDAC creates an immune-suppressive tumor microenvironment to escape immune-mediated eradication. Regulatory T (Treg) cells and myeloid-derived suppressor cells (MDSC) are critical components of the immune-suppressive tumor microenvironment. Shifting from tumor escape or tolerance to elimination is the major challenge in the treatment of PDAC.

**Results:**

In a mathematical model, we combine distinct treatment modalities for PDAC, including 5-FU chemotherapy and anti- CD25 immunotherapy to improve clinical outcome and therapeutic efficacy. To address and optimize 5-FU and anti- CD25 treatment (to suppress MDSCs and Tregs, respectively) schedule in-silico and simultaneously unravel the processes driving therapeutic responses, we designed an in vivo calibrated mathematical model of tumor-immune system (TIS) interactions. We designed a user-friendly graphical user interface (GUI) unit which is configurable for treatment timings to implement an in-silico clinical trial to test different timings of both 5-FU and anti- CD25 therapies. By optimizing combination regimens, we improved treatment efficacy. In-silico assessment of 5-FU and anti- CD25 combination therapy for PDAC significantly showed better treatment outcomes when compared to 5-FU and anti- CD25 therapies separately. Due to imprecise, missing, or incomplete experimental data, the kinetic parameters of the TIS model are uncertain that this can be captured by the fuzzy theorem. We have predicted the uncertainty band of cell/cytokines dynamics based on the parametric uncertainty, and we have shown the effect of the treatments on the displacement of the uncertainty band of the cells/cytokines. We performed global sensitivity analysis methods to identify the most influential kinetic parameters and simulate the effect of the perturbation on kinetic parameters on the dynamics of cells/cytokines.

**Conclusion:**

Our findings outline a rational approach to therapy optimization with meaningful consequences for how we effectively design treatment schedules (timing) to maximize their success, and how we treat PDAC with combined 5-FU and anti- CD25 therapies. Our data revealed that a synergistic combinatorial regimen targeting the Tregs and MDSCs in both crisp and fuzzy settings of model parameters can lead to tumor eradication.

**Supplementary Information:**

The online version contains supplementary material available at 10.1186/s12885-021-08770-z.

## Introduction

Pancreatic cancer is the seventh leading cause of mortality related to cancer around the world [1] and the fourth leading cause in the United States [2], and it is predicted to be the second leading cause by 2030 [3]. Pancreatic ductal adenocarcinoma (PDAC) constitutes 85% of histological diagnoses of pancreatic cancer, and this subtype mostly emerges from the exocrine glands of the neck and head of the pancreas [4]. PDAC has a very poor prognosis with the lowest surveillance among all cancers, a five-year overall relative survival rate of 8% [1]. This disappointing surveillance is due to a delay in diagnosis of this disease because of having no specific symptom; thus, patients are usually diagnosed with metastasis or an advanced unresectable mass [5]. Additionally, the only current curative therapy for this disease is surgery, but only 20% of patients have locally resectable mass when diagnosed [6, 7]. So, various therapeutic modalities like immunotherapy, neoadjuvant chemotherapy, radiotherapy, and surgery are performed to improve surveillance and alleviate the patient’s discomfort, but there is no curative approach for an advanced PDAC already [8]. Recent findings showed that modulating the effector cells in the tumor microenvironment by immunotherapy or chemotherapy would lead to impressive clinical and experimental therapeutic effects on PDAC [9–12]. Besides, some specific features of PDAC, like the existence of many immunosuppressive mediators in its microenvironment that are surrounded by a dense stroma cause the tumor to physically block the penetration of the drug; thus, this issue emphasizes the promising efficacy of immunotherapy specifically when combined with chemotherapy [13]. Till now, the experimental studies showed the beneficial therapeutic effect of Anti-CD25 immunotherapy targeting Treg cells [14–16] as well as 5-fluorouracil (5-FU) chemotherapy targeting MDSCs [12, 17, 18] on PDAC, but to our knowledge, there is no experimental study on the efficacy of Anti-CD25 and 5-FU combination therapy for PDAC. Also, several mathematical models have been conducted on the anti-tumor effect of Anti-CD25 and 5-FU. Shariatpanahi et al. designed an ODE model to simulate the effect of MDSC depletion by 5-FU on tumor-immune system dynamics and to evaluate the effect of replication of this treatment on tumor degradation. In their study, they designed a simulation framework to capture the dynamics of tumor cells, MDSC, CTL, and NK cells with and without 5-FU treatment. Their study using in silico assessment of 5-FU treatment proposed a testable hypothesis in vivo/in vitro environments [19]. In another study, Loizides et al. constructed an ODE model to capture the tumorigenesis process and tumor interactions with the immune system. They also modeled 5-FU therapy using a two compartmental pharmacokinetic/pharmacodynamics model. Their Gompertz model simulated overall characteristics of the inherent variability of in vivo tumor growth rates and 5-FU killing effects that was observed in the de novo animal cancer model and predicted by mathematical modeling [20]. Montiel et al. developed a mathematical model to interrogate the effects of immunotherapy using dendritic cells (DCs) on tumor-immune system interactions. Their model consists of five delay differential equations that are calibrated by experimental data and are used to test different immunotherapy protocols. By in silico assessment of DC therapy, they suggest that changing the infusion time and using more doses of DCs causes more degradation of tumor cells [21].

Although various mathematical model analyses were conducted to determine the efficacy of immunotherapy or chemotherapy for pancreatic cancer separately [22–25], the effect of anti-CD25 immunotherapy in combination with 5-FU chemotherapy on pancreatic ductal adenocarcinoma has not assessed so far. Therefore, in the present study, we constructed a mathematical model based on ordinary differential equations (ODEs) to describe the dynamic interactions among dominant cells and their cytokines in the pancreatic tumor microenvironment during different phases of treatments and provided a quantitative prediction of anti-CD25 and 5-FU efficacy for pancreatic ductal adenocarcinoma.

## Model development

### Biological concept

**Fig. 1:**
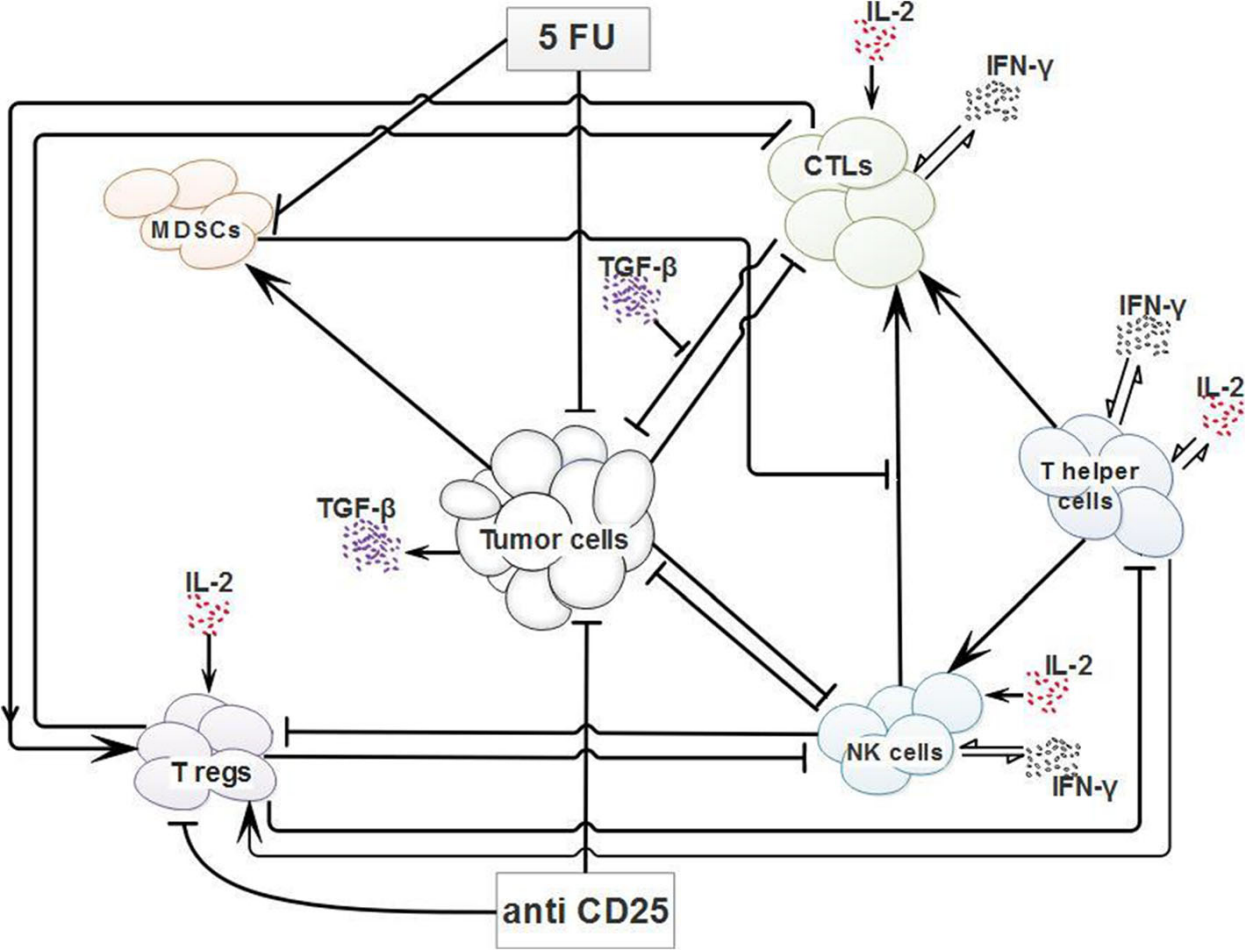
Conceptual model of tumor-immune system interactions. The arrows depict activation/induction and blocked arrows indicate blocking/inhibiting

Figure 1 demonstrates a simplified biological concept of the immunosuppressive mechanisms and antitumor activities mediated by various effector cells in the tumor microenvironment as well as the influence of 5-FU chemotherapy and anti-CD25 immunotherapy on these interactions which are used for mathematical modeling. Besides, we considered the following biological assumptions in our model with regards to the previous studies [19, 26–34].

1. Tumor cells have the logistic growth in deprivation of the immune system [28].
2. Tumor cells stimulate the activation of cytotoxic T lymphocytes [19].
3. In the tumor microenvironment, NK cells also stimulate the activation of cytotoxic T lymphocytes [19].
4. NK cells and activated cytotoxic T lymphocytes induce tumor cell regression [19, 29].
5. The activity of NK cells and cytotoxic T lymphocytes declines over time after encountering tumor cells [19, 30].
6. Tumor cells also recruit MDSCs and stimulate their proliferation in the tumor microenvironment [19, 31].
7. MDSCs suppress cytotoxic T lymphocyte activation mediated by NK cells in the tumor microenvironment [19, 26].
8. Treg cells that are increased in the tumor microenvironment suppress the proliferation of NK cells, cytotoxic T lymphocytes, and T helper cells [32].
9. Low doses of 5-FU chemotherapy inhibit tumor progression through the deactivation of MDSCs [19, 27].
10. Anti-CD25 immunotherapy inhibits tumor progression through the depletion of Treg cells [34].

### Methodology and experiment setting

We confirm that no animals were involved in the present study. In this study, we proposed a mathematical model that is calibrated by in vivo data of two studies. Wu et al. [35] and Pu et al. [23] presented in vivo data for change in tumor size during 5-FU chemotherapy and anti-CD25 immunotherapy, respectively. The experiments of Wu et al. study in the control group (no treatment) and 5-FU therapy group are as follows. Control group: C57BL/6 mice were subcutaneously implanted with Panc02 cells (6 × 10^5^ per mouse) in the right flank and tumor size is recorded every 5 days up to 25 days beginning 5 days after tumor inoculation. 5-FU therapy group: this group is similar to the control group, except that on the first to fourth days, at each day a single dose of 5-FU (30 mg/kg) was injected. We used the trend of tumor size recorded over time (data records are extracted from figures by *WebPlotDigitizer* tool [36]) to estimate some parameters of the model which are describing the effect of 5-FU therapy in pancreatic cancer. In the experiment of Pu et al., C57BL/6 mice were subcutaneously inoculated with Panc02 cells (2 × 10^6^ per mouse) and receiving CD25 antibody (50 μg per mouse) twice a week for 3 weeks beginning 3 days after tumor induction. The trend of tumor size measured in the Pu et al. study was used to estimate parameters reflecting the effect of anti-CD25 therapy. Both studies of Wu et al. and Pu et al. were performed on a specific cell line (Panc02) and the same mice (C57BL/6 mice). We used the data of these experiments to evaluate the effect of the combinatorial manner of 5-FU and anti-CD25 therapies on pancreatic cancer. The parameterized mathematical model using control, 5-FU, and anti-CD25 data sets, reproduced the published in vivo data of both studies to describe tumor-immune cell interactions through 5-FU and anti-CD25 therapy. This mathematical model enables us to investigate the interaction effects of treatments (synergistic, additive, or antagonistic effect), optimize time schedules of treatments, and deepen our understanding of complex dynamics and regulatory mechanisms in the tumor microenvironment.

### General characteristics of the mathematical model used for anticipating the therapeutic effects of 5FU and anti-CD25 on pancreatic cancer

The mathematical model of the tumor-immune system of this study is constructed using ODEs to describe the dynamics of interactions between pancreatic cancer cells and major innate and adaptive immune system components includes cytotoxic T lymphocytes (CTLs), natural killer (NK) cells, myeloid-derived suppressor cells (MDSCs), TCD4+ cells (T helper), T regulatory cells (Tregs) and cytokines e.g. interleukin-2 (IL-2), interferon-gamma (IFN-γ) and transforming growth factor-beta (TGF-β). The model considered anti-CD25 monotherapy as well as low-dose 5-fluorouracil (5-FU) chemotherapy to predict their efficacy for pancreatic cancer. The anti-CD25 and 5-FU treatments modeling techniques include the techniques that were used in the studies of Dan-Hua HE et al. [34] and SP Shariatpanahi et al. [19], respectively. The mathematical model is constructed using nine ODEs (eqs. 1–9) that each equation describes the change in population/concentration of cells/cytokines. All model variables including *C, N, T, M, H, R, I, F, S,* and *antiCD25* are time-dependent; hence, for example, for the sake of brevity, we have written *C* instead of *C(t)*.

1 $$ \frac{dC}{dt}={a}_1C\ \log \left(\frac{C_{max}}{C}\right)-{b}_1N\frac{\mathrm{C}}{1+\frac{1}{l_1}{C}^{\frac{1}{3}}}-\frac{\frac{c_1T\mathrm{C}}{1+\frac{1}{l_1}{C}^{\frac{1}{3}}}}{\left(\frac{1+{e}_1R}{1+{d}_1{(antiCD25)}^2{R}^3}\right)\left(1+{f}_1S\right)}-{g}_1C-{h}_1\log \left(1+{k}_1\mathit{\operatorname{sign}}(antiCD25){\int}_0^t antiCD25\left(\tau \right)C\left(\tau \right) d\tau \right) $$

Pancreatic cancer cell (C): In eq. (1), $\frac{dC}{dt}$ describes the time derivative of cancer cells, and this equation represents the dynamics of pancreatic cancer cells. The first term on the right side of the equation describes the Gompertzian growth of cancer cells, where *a*_1_ is the growth rate of cancer cells in absence of treatment and *C*_*max*_ is the carrying capacity or the maximum size of the tumor in this model [19]. The second and third terms represent the NK-mediated and CTL-mediated killing of accessible tumor cells ($\frac{\mathrm{C}}{1+\frac{1}{l_1}{C}^{\frac{1}{3}}}$) to NKs and CTLs, respectively, and by the depth of access *l*_1_ [19]. Term $\frac{\mathrm{C}}{1+\frac{1}{l_1}{C}^{\frac{1}{3}}}$ represents the surface layers of tumor cells in tumor mass [19]. The maximum rates of NK-mediated and CTL-mediated tumor cell killing are parameters *b*_1_ and *c*_1_, respectively [19]. The terms $\left(\frac{1+{e}_1R}{1+{d}_1{(antiCD25)}^2{R}^3}\right)$ and (1 + *f*_1_*S*) in the denominator of the third term describe the inhibitory effects of Tregs and TGF-β on CTL-mediated cancer cell killing. The proportional parameter *d*_1_ regulates the weight (intensity) of anti-CD25 monotherapy and parameters *e*_1_ and *f*_1_ reflect the measure of the inhibitory effect of Tregs and TGF-β on CTL cytotoxicity, respectively [34]. The denominator of term $\left(\frac{1+{e}_1R}{1+{d}_1{(antiCD25)}^2{R}^3}\right)$ represents the Treg depletion by anti-CD25 antibody clone that it improves CTL cytotoxicity by inhibition of Tregs. The powers 2 and 3 for antiCD25 treatment and Treg cells (*R*) were used to adjust model for better calibration and parametrization. We assumed that chemotherapy using low dose 5-FU and monotherapy using anti-CD25 directly impacts the pancreatic tumor volume that is reflected in the fourth and fifth terms of eq. (1), respectively. The parameter *g*_1_ represents the apoptotic rate of tumor cells by 5-FU and the parameters *h*_1_ and *k*_1_ are the apoptotic rate of pancreatic cancer cells by anti-CD25 treatment. The function *sign*(*antiCD*25) is used to indicate whether or not the anti-CD25 monotherapy is involved; therefore, this function is one when anti-CD25 treatment is applied and is zeros otherwise [37, 38]. Term *antiCD25(τ)* presents the administration pattern of anti-CD25 therapy [34]. Term ${\int}_0^t antiCD25\left(\tau \right)C\left(\tau \right) d\tau$ represents the total dose of *antiCD25*(τ), 0 ≤ *τ* ≤ *t* injected until time *t*. This integral term computes the total dose of anti-CD25 and include this term in a logarithmic function as a non-monotonic function to simulate the decay of affected cancer cell by treatment. Actually, this term indicates that the population of cancer cells at time *t* is depended on total dose of injected antiCD25(τ) at time *τ* ≤ *t.* The use of integral terms and non-monotonic functions for simulation of kinetic-dynamics of biological phenomena is convenient, for example, in a study, integral terms were used to simulate the dynamics of cytokines [39]. Also, in other study, Islam et al. used sliding model approach to determine anticancer agent dosing and they used non-monotonic functions to simulate anticancer therapeutic effect on cancer cells [40].

2 $$ \frac{dN}{dt}={a}_2-{b}_2N+\frac{{c^{\bullet}}_2 IN}{d_2+I}+\frac{e_2 FN}{f_2+F}-\frac{g_2N\mathrm{C}}{1+\frac{1}{l_1}{C}^{\frac{1}{3}}}-{h}_2 RN $$

Natural killer cell (N): Eq. (2) describes the dynamic of NKs (N). The first and second terms on the right side of the equation represent the constant production rate of NKs in bone marrow with parameter *a*_2_ and normal death rate of NKs with an exponential rate *b*_2_, respectively [19]. The third and fourth terms describe the IL-2 mediated and IFN-γ mediated stimulation of NK cells, respectively, that are modeled in the simplified version of the model (eq. 14) by a Michaelis-Menten form with parameters *c*_2_, ${d}_2\frac{\tau_1}{\alpha_1}$, *e*_2_ and *f*_2_ [34]. The fifth term represents the inactivation of NK cells by interacting with accessible tumor cells with maximum inactivation rate *g*_2_ [19]. The last term describes Treg-mediated NK cell killing (inactivation) in a granzyme-B-dependent fashion with a constant rate *h*_2_ [34].

3 $$ \frac{dT}{dt}=-{a}_3T+\frac{b_3T{C}^2}{c_{3+}{C}^2}+\frac{d_3N\mathrm{C}}{1+\frac{1}{l_1}{C}^{\frac{1}{3}}}\left(\frac{1-{m}_3}{1+{n}_3{\left(M-{p}_3\right)}^2}+{m}_3\right)-\frac{e_3T\mathrm{C}}{1+\frac{1}{l_1}{C}^{\frac{1}{3}}}+\frac{{f^{\bullet}}_3 IT}{g_3+I}+\frac{h_3 FT}{k_3+F}-{l}_3 RT $$

Cytotoxic T lymphocytes (T): Eq. (3) describes the dynamics of CTLs as the major component of the adaptive immune system. The first term on the right side of the equation stands for the death of CTLs with an exponential rate *a*_3_ [19]. The second term describes the tumor recruitment of CTLs that has a Michaelis-Menten form with parameters *b*_3_ and *c*_3_ [19]. The third term shows the activation of CTLs as a result of interactions of NKs and accessible tumor cells with a constant rate *d*_3_ [19]. Also, MDSCs negatively regulate this interaction to prevent CTL activation. The parameter *p*_3_ is the normal population of MDSCs when there is no tumor and parameters *m*_3_ and *n*_3_ are effectiveness factors and proportional parameters related to MDSC-mediated inhibition of CTL activation [19]. The fourth term represents the inactivation rate of interacting CTLs with accessible tumor cells with a constant rate *e*_3_ [19]. The fifth and sixth terms show the stimulatory effects of cytokines IL-2 and IFN-γ on CTL proliferation/activation that they are modeled by Michaelis-Menten form with parameters *f*^●^_3_, *g*_3_, *h*_3_, *k*_3_ and with parameters *f*_3_, ${g}_3\frac{\tau_1}{\alpha_1}$, *h*_3_, *k*_3_ (eq. 15) [34]. The last term represents Treg-mediated CTL inhibition with a constant rate *l*_3_ [34].

4 $$ \frac{dM}{dt}={a}_4-{b}_4M+\frac{c_4C}{d_4+C} $$

Myeloid derives suppressor cell (M): Eq. (4) describes the dynamics of tumor-induced MDSCs. The first term on the right side of the equation shows the constant recruitment rate *a*_4_ of bone marrow produced MDSCs to the spleen [19]. The second term represents the death rate of MDSCs with a constant rate (*b*_4_) in normal conditions or during 5-FU treatment [19]. The third term describes the tumor-induced expansion of MDSCs with parameters *c*_4_ and *d*_4_ [19].

5 $$ \frac{dH}{dt}={a}_5-{b}_5H+\frac{{c^{\bullet}}_5 IH}{d_5+I}+\frac{e_5 FH}{f_5+F}-{g}_5 RH $$

T helper cells (H): Eq. (5) represents the dynamics of T helper cells. The first term on the right side of eq. (5) describes the production rate (*a*_5_) of T helper cells in the thymus [34]. The second term describes the exponential degradation rate (*b*_5_) of T helper cells [34]. The third and fourth terms show the activation rate of T helper cells by IL-2 and IFN-γ, respectively, that are modeled by a Michaelis-Menten form with parameters *c*^●^_5_, *d*_5_, *e*_5_, *f*_5_ and parameters *c*_5_, ${d}_5\frac{\tau_1}{\alpha_1}$, *e*_5_ and *f*_5_ (the third and fourth terms of eq. 17) [34]. The last term models the inactivation/degradation of T helper cells by Tregs with a constant rate *g*_5_ [34].

6 $$ \frac{dR}{dt}={a}_6-{b}_6R+{c}_6T+{d}_6H+\frac{{e^{\bullet}}_6 IR}{f_6+I}-{g}_6 NR-{h}_6(antiCD25)R $$

Regulatory T cell (R): eq. (6) describes the dynamic of Tregs as an important component of the immune system for the induction of peripheral tolerance. The first term on the right side of the equation describes the constant production rate of Tregs from their origin in the thymus and peripheral (*a*_6_) [34]. The second term shows the exponential death rate of Tregs (*b*_6_) [34]. The third and fourth terms show that Tregs originate from both TCD8+ and T helper cells and the population of Tregs increases with the sum of these two terms (*c*_6_*T* + *d*_6_*H*) that is proportional to CTLs population with a constant rate *c*_6_ and T helper population with a constant rate *d*_6_ [34]. The fifth term describes the IL-2-mediated growth rate of Tregs that is modeled by a Michaelis-Menten form with parameters *e*^●^_6_, *f*_6_, and parameters *e*_6_ and ${f}_6\frac{\tau_1}{\alpha_1}$ (the fifth term in eq. 18) [34]. The sixth term describes that NKs degrade Treg cells with a constant rate *g*_6_ and the last term models anti-CD25-mediated Treg depletion with a constant rate *h*_6_ [34].

7 $$ \frac{dI}{dt}={\alpha}_1H-{\tau}_1I $$

Interleukin-2 (I): Eq. (7) represents the dynamic of IL-2. The first term on the right side describes the IL-2 secretion from T helper with a constant rate *α*_1_, and the second term describes the degradation with a rate *τ*_1_ [34].

8 $$ \frac{dF}{dt}={\beta}_1T+{\beta}_2N+{\beta}_3H-{\tau}_2F $$

Interferon-gamma (F): Eq. (8) shows the dynamic of IFN-γ. According to terms 1–3 of eq. (8), IFN-γ is produced by CD8 + T cells, NK cells and T helper cells with constant rates *β*_1_, *β*_2_ and *β*_3_, respectively [34]. The last term of the equation describes the natural death rate of IFN-γ based on its half-life [34].

9 $$ \frac{dS}{dt}={\lambda}_1C-{\tau}_3S $$

Transforming growth factor-beta (S): Eq. (9) describes the dynamics of TGF-β which is produced by cancer cells with a constant rate *λ*_1_ and is degraded with a rate *τ*_3_ based on its half-life [34].

The changes in concentration of cytokines compared to the population of cells occur on a very short time scale; therefore, we can use quasi-steady-state approximations (eqs. 10–12) for cytokine concentrations to simplify our model.

10 $$ \frac{dI}{dt}=0;\kern0.75em I=\frac{\alpha_1}{\tau_1}H $$

11 $$ \frac{dF}{dt}=0;\kern1em F=\frac{\beta_1}{\tau_2}T+\frac{\beta_2}{\tau_2}N+\frac{\beta_3}{\tau_2}H $$

12 $$ \frac{dS}{dt}=0;\kern0.5em S=\frac{\lambda_1}{\tau_3}C $$

By substituting eqs. 10–12 into eqs. 1–6, the simplified model, as described using eqs. 13–18, is achieved. Finally, the simplified model of the tumor-immune system includes 6 ODEs and 65 kinetic parameters. Some kinetic parameters are estimated based upon the experimental data of 5-FU chemotherapy and anti-CD25 monotherapy in a murine model of PDAC in the C57/BL6 mouse using Panc02 cell line.

13 $$ \frac{dC}{dt}={a}_1C\ \log \left(\frac{C_{max}}{C}\right)-{b}_1N\frac{\mathrm{C}}{1+\frac{1}{l_1}{C}^{\frac{1}{3}}}-\frac{\frac{c_1T\mathrm{C}}{1+\frac{1}{l_1}{C}^{\frac{1}{3}}}}{\left(\frac{1+{e}_1R}{1+{d}_1{(antiCD25)}^2{R}^3}\right)\left(1+{f}_1\frac{\lambda_1}{\tau_3}C\right)}-{g}_1C-{h}_1\log \left(1+{k}_1\mathit{\operatorname{sign}}(antiCD25){\int}_0^t antiCD25\left(\tau \right)C\left(\tau \right) d\tau \right) $$

14 $$ \frac{dN}{dt}={a}_2-{b}_2N+\frac{c_2 HN}{d_2\frac{\tau_1}{\alpha_1}+H}+\frac{e_2\left(\frac{\beta_1}{\tau_2}T+\frac{\beta_2}{\tau_2}N+\frac{\beta_3}{\tau_2}H\right)N}{f_2+\frac{\beta_1}{\tau_2}T+\frac{\beta_2}{\tau_2}N+\frac{\beta_3}{\tau_2}H}-{g}_2N\frac{\mathrm{C}}{1+\frac{1}{l_1}{C}^{\frac{1}{3}}}-{h}_2 RN $$

15 $$ \frac{dT}{dt}=-{a}_3T+\frac{b_3T{C}^2}{c_{3+}{C}^2}+\frac{d_3N\mathrm{C}}{1+\frac{1}{l_1}{C}^{\frac{1}{3}}}\left(\frac{1-{m}_3}{1+{n}_3{\left(M-{p}_3\right)}^2}+{m}_3\right)-{e}_3T\frac{\mathrm{C}}{1+\frac{1}{l_1}{C}^{\frac{1}{3}}}+\frac{f_3 HT}{g_3\frac{\tau_1}{\alpha_1}+H}+\frac{h_3\left(\frac{\beta_1}{\tau_2}T+\frac{\beta_2}{\tau_2}N+\frac{\beta_3}{\tau_2}H\right)T}{k_3+\left(\frac{\beta_1}{\tau_2}T+\frac{\beta_2}{\tau_2}N+\frac{\beta_3}{\tau_2}H\right)}-{l}_3 RT $$

16 $$ \frac{dM}{dt}={a}_4-{b}_4M+\frac{c_4C}{d_4+C} $$

17 $$ \frac{dH}{dt}={a}_5-{b}_5H+\frac{c_5{H}^2}{d_5\frac{\tau_1}{\alpha_1}+H}+\frac{e_5\left(\frac{\beta_1}{\tau_2}T+\frac{\beta_2}{\tau_2}N+\frac{\beta_3}{\tau_2}H\right)H}{f_5+\frac{\beta_1}{\tau_2}T+\frac{\beta_2}{\tau_2}N+\frac{\beta_3}{\tau_2}H}-{g}_5 RH $$

18 $$ \frac{dR}{dt}={a}_6-{b}_6R+{c}_6T+{d}_6H+\frac{e_6 HR}{f_6\frac{\tau_1}{\alpha_1}+H}-{g}_6 NR-{h}_6(antiCD25)R $$

### The goodness of fit assessment

For model fitting, we used a Genetic algorithm (GA) method to estimate model parameters. The cost function of GA is a normalized root-mean-square error (NRMSE), defined as follow:

T_1_, T_2_, …, T_5_ were the tumor volumes measuring at time points of t_1_, t_2_, …, t_5_. Also, ${\hat{\mathrm{T}}}_1,{\hat{\mathrm{T}}}_2,\dots, {\hat{\ \mathrm{T}}}_5$ were the predicted tumor volumes by the mathematical model at the same time points. The formulation of NRMSE is defined by $NRMSE=\sqrt{\frac{\sum \limits_{i=1}^5{\left({T}_i-{\hat{T}}_i\right)}^2}{{T_1}^2}}$. Actually our model predicts the populaion of tumor cells that we convert it into tumor volume by considering the volume of a single cancerous cell is 10^−6^*mm*^3^ [41, 42].

### Uncertainty analysis

There are two types of uncertainty in biological network modeling including fuzzy uncertainty and random uncertainty [43]. Due to imprecise, missing, or incomplete experimental data, the kinetic parameters of computational models are uncertain, and assigning a fuzzy uncertain number instead of crisp value to kinetic parameters seems a better choice [36–45]. Also, the effect of random uncertainty on dynamics of model constituents and robustness of model against perturbations can be determined by sensitivity analysis. For this aim, we used two different methods including partial rank correlation coefficient (PRCC) [46] and elementary effect (EE) test [47]. In the results section, we assess the effect of the fuzzy uncertainty of parameters on the dynamics of tumor cells in the presence and absence of treatments. Also, the results of model robustness and the relation between dynamics of cells/cytokines and kinetic parameters are computed by sensitivity analyses that are provided in the results section.

## Results

### Model calibration for the prediction of the dynamics of pancreatic tumor cells in control case, 5-FU and anti-CD25 therapies

We used GA to estimate model parameters in no treatment case to predict the dynamics of tumor-immune system constituents in the control group. We recruited the experimental data of the control group of study [35] (the recorded Panc02 tumor size on days 5, 10, 15, 20, and 25 after tumor inoculation on day 0) for model fitting and the resulted NRMSE was 0.1107. To estimate model parameters (*g*_1_***,****b*_4_) related to the inhibitory effect of 5-FU therapy on pancreatic tumor cells, we used the data of the 5-FU treatment group of study [35] (the recorded Panc02 tumor cell population on days 5, 10, 20, and 25 after tumor inoculation on day 0, with regarding 5-FU therapy is carried out on days 1, 2, 3 and 4 after tumor inoculation). Similarly, the model was fitted to in vivo data by NRMSE = 0.1085. Finally, we used the experimental data of the anti-CD25 treatment group of study [23] (the recorded Panc02 tumor cell population on days 7, 14, 21, 28, and 35 after tumor inoculation on day 0, with regarding that anti-CD25 therapy is carried out on days 3, 6, 10, 13, 17 and 20 after tumor inoculation) to estimate model parameters (*d*_1_, *h*_1_, *k*_1_, *h*_6_) related to the effect of anti-CD25 therapy on pancreatic tumor cells. The model was fitted to in vivo data by NRMSE of 0.0928.

**Fig. 2:**
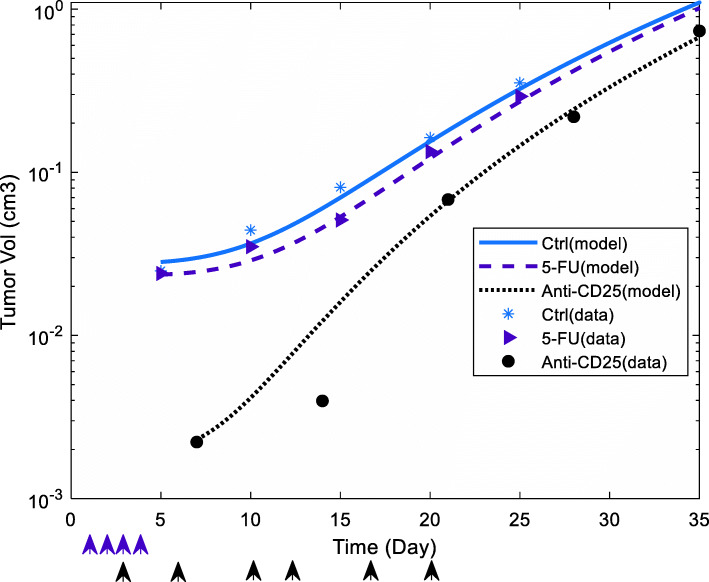
Data fitting. The blue line shows predicted Panc02 tumor volume dynamics in no treatment case and blue stars are records of Panc02 tumor volume on days 5, 10, 15, 20, and 25 in the control group (Panc02 tumor inoculation is carried out on day 0 and normalized root mean square error (NRMSE = 0.1107) is used as a measure of goodness of fit). The purple dashed line shows predicted dynamics of Panc02 tumor volume and purple ‘>‘are data points gathered from experimental data in the 5-FU treatment group (5-FU therapy is carried out on days 1, 2, 3, and 4 after Panc02 tumor injection on day 0, and data record is carried out on days 5, 10, 15, 20 and 25, and NRMSE of 0.1085 is computed to assess model fitting). The black dotted line shows predicted dynamics of Panc02 tumor cells and black ‘circles’ are data points gathered from in vivo experiments in the anti-CD25 treatment group (anti-CD25 therapy is carried out on days 3, 6, 10, 13, 17, and 20 after tumor inoculation on day 0 and data record is carried out on days 7, 14, 21, 28 and 35, and NRMSE of 0.0928 is computed to assess model fitting)

The model parameters, their description, units, and references are provided in Table 1. As shown in Fig. 2, model fitting is carried out and predicted dynamics of tumor population by the parameterized model is fitted to tumor sizes measured in the control group, 5-FU therapy group, and anti-CD25 therapy group.

**Table 1:** Summary of parameter values

Parameter	Value	Definition	Units	References
***a***_**1**_	4.3992 × 10^−2^	Panc02 tumor growth rate	$\frac{1}{day}$	Estimated
***C***_***max***_	1 × 10^10^	Maximum sustainable tumor cell population	*cell*	[19]
***b***_**1**_	3.23 × 10^−7^	NK-mediated tumor cell kill rate	$\frac{1}{cell\times day}$	[19]
***c***_**1**_	1.1 × 10^−7^	CTL-mediated tumor cell kill rate	$\frac{1}{cell\times day}$	[19]
***d***_**1**_	2 × 10^−34^	Proportional parameter of anti-CD25 treatment for inhibition of anti-immune effects of Treg on CTL-mediated tumor cell killing	$\frac{1}{cell^3}$	Estimated
***e***_**1**_	0.345	Rate of the suppressive effect of Treg on CTL-mediated tumor cell killing	$\frac{1}{cell}$	[34]
***f***_**1**_	0.286	Rate of the suppressive effect of TGF-β on CTL-mediated tumor cell killing	$\frac{ml}{ng}$	[34]
***g***_**1**_	3.5 × 10^−2^	The apoptosis rate of Panc02 tumor cells by low-dose 5-FU treatment	$\frac{1}{day}$	Estimated
***h***_**1**_	2.5 × 10^5^	Proportional parameter of tumor inhibition rate by Treg depletion through anti-CD25 treatment	$\frac{cell}{day}$	Estimated
***k***_**1**_	1 × 10^−15^	Proportional parameter of tumor inhibition rate by Treg depletion through anti-CD25 treatment	$\frac{1}{cell}$	Estimated
***l***_**1**_	100	Depth of access of immune cells to the tumor mass	${cell}^{\frac{1}{3}}$	[19]
***a***_**2**_	1.4 × 10^4^	Constant generation source of NK cells	$\frac{cell}{day}$	[19]
***b***_**2**_	4.12 × 10^−2^	The exponential death rate of NK cells	$\frac{1}{day}$	[19]
***c***_**2**_	0.125	Maximum of IL-2-mediated NK cell growth	$\frac{1}{day}$	[34]
***d***_**2**_	0.3	Steepness coefficient of the IL-2-mediated NK cell growth rate	$\frac{ng}{ml}$	[34]
***e***_**2**_	0.125	Maximum of IFN-γ mediated NK cell growth rate	$\frac{1}{day}$	[34]
***f***_**2**_	0.3	The steepness coefficient of the IFN-γ-mediated NK cell growth rate	$\frac{ng}{ml}$	[34]
***g***_**2**_	1 × 10^−9^	Inactivation rate of NK cells by tumor cells	$\frac{1}{day}$	Estimated close to the value reported in [19]
***h***_**2**_	1 × 10^−10^	Suppression rate of NK cells by Tregs.	$\frac{1}{cell\times day}$	[34]
***a***_**3**_	2 × 10^−2^	The exponential death rate of CTLs	$\frac{1}{day}$	[19]
***b***_**3**_	8 × 10^−2^	Maximum tumor-mediated CTL recruitment rate	$\frac{1}{day}$	Estimated close to the value reported in [19]
***c***_**3**_	2.02 × 10^14^	Steepness coefficient of the tumor-mediated CTL recruitment curve	*cell*^2^	Estimated with regarding the value reported in [19]
***d***_**3**_	1.1 × 10^−7^	CTL stimulation rate by tumor-NK cells interactions	$\frac{1}{cell\times day}$	[19]
***e***_**3**_	1.5 × 10^−10^	Inactivation rate of CTLs by tumor cells	$\frac{1}{cell\times day}$	Estimated close to the value reported in [19]
***f***_**3**_	125 × 10^−5^	Maximum of IL-2-mediated CTL growth rate	$\frac{1}{day}$	Estimated with regarding the value reported in [34]
***g***_**3**_	0.3	The steepness coefficient of the IL-2-mediated CTL growth rate	$\frac{ng}{ml}$	[34]
***h***_**3**_	12.5 × 10^−2^	Maximum of IFN-γ-mediated CTL growth rate	$\frac{1}{day}$	[34]
***k***_**3**_	0.3	The steepness coefficient of the IFN-γ-mediated CTL growth rate	$\frac{ng}{ml}$	[34]
***l***_**3**_	1 × 10^−10^	Suppression rate of CTLs by Tregs	$\frac{1}{cell\times day}$	[34]
***p***_**3**_	2.5 × 10^6^	The normal number of splenic MDSCs in C57/BL6 mice	*cell*	[19]
***m***_**3**_	18 × 10^−2^	Minimal CTL proliferation factor induced by inhibition of MDSCs	*---*	[19]
***n***_**3**_	6 × 10^−3^	Parameter for MDSC-induced inhibition of CTL proliferation	$\frac{1}{cell^2}$	[19]
***a***_**4**_	1.25 × 10^6^	Normal MDSC production rate	$\frac{cell}{day}$	[19]
***b***_**4**_	3.25 × 10^−2^	MDSCs normal death rate	$\frac{1}{day}$	Estimated with regarding the value reported in [19]
***b***_**4**_	8 × 10^−2^	MDSCs death rate during 5-FU treatment	$\frac{1}{day}$	Estimated with regarding the value reported in [19]
***c***_**4**_	0.7 × 10^7^	MDSC expansion coefficient in Panc02 tumor-bearing mice	$\frac{cell}{day}$	[19]
***d***_**4**_	1 × 10^10^	Steepness coefficient of the tumor-mediated MDSC production curve	*cell*	[19]
***a***_**5**_	3.6 × 10^5^	The production rate of T helper cells in the thymus	$\frac{cell}{day}$	[34]
***b***_**5**_	1.2 × 10^−3^	The exponential death rate of T helper cells based on the half-life	$\frac{1}{day}$	[34]
***c***_**5**_	*0.125*	Maximum IL-2-mediated T helper cell proliferation rate	$\frac{1}{day}$	[34]
***d***_**5**_	0.3	Steepness coefficient of the IL-2-mediated T helper cell proliferation curve	$\frac{ng}{ml}$	[34]
***e***_**5**_	*0.125*	Maximum IFN-γ-mediated T helper cell proliferation rate	$\frac{1}{day}$	[34]
***f***_**5**_	0.3	Steepness coefficient of the IFN-γ-mediated T helper cell proliferation curve	$\frac{ng}{ml}$	[34]
***g***_**5**_	1 × 10^−10^	Suppression rate of T helper cells by Tregs	$\frac{1}{cell\times day}$	[34]
***a***_**6**_	5.6 × 10^5^	The constant production rate of Tregs	$\frac{cell}{day}$	[34]
***b***_**6**_	2.3 × 10^−2^	The exponential death rate of Tregs based on the half-life	$\frac{1}{\ day}$	[34]
***c***_**6**_	2 × 10^−4^	Treg origination rate from CTLs	$\frac{1}{\ day}$	[34]
***d***_**6**_	4 × 10^−4^	Treg origination rate from T helper cells	$\frac{1}{\ day}$	[34]
***e***_**6**_	0.125	Maximum IL-2-mediated growth rate of Tregs	$\frac{1}{day}$	[34]
***f***_**6**_	0.3	Steepness coefficient of the IL-2-mediated Treg growth curve	$\frac{ng}{ml}$	[34]
***g***_**6**_	1 × 10^−11^	NK-mediated Treg degradation constant rate	$\frac{1}{cell\times day}$	[34]
***h***_**6**_	1.5 × 10^−11^	Constant inhibition rate of Tregs by anti-CD25 treatment	$\frac{1}{\ day}$	[34]
$\frac{{\boldsymbol{\tau}}_{\mathbf{1}}}{{\boldsymbol{\alpha}}_{\mathbf{1}}}$	2.2483 × 10^11^	The natural death rate of IL-2 based on its half-life/ constant production rate of IL-2 by T helper cells	$\frac{cell\times ml}{ng}$	[34]
$\frac{{\boldsymbol{\beta}}_{\mathbf{1}}}{{\boldsymbol{\tau}}_{\mathbf{2}}}$	4.4691 × 10^−13^	constant production rate of IFN-γ by CTLs/ Natural death rate of IFN-γ based on its half-life	$\frac{ng}{ml\times cell}$	[34]
$\frac{{\boldsymbol{\beta}}_{\mathbf{2}}}{{\boldsymbol{\tau}}_{\mathbf{2}}}$	4.4691 × 10^−13^	The secretion rate of IFN-γ by NK cells/degradation rate of IFN-γ based on its half-life	$\frac{ng}{ml\times cell}$	[34]
$\frac{{\boldsymbol{\beta}}_{\mathbf{3}}}{{\boldsymbol{\tau}}_{\mathbf{2}}}$	4.4691 × 10^−13^	The secretion rate of IFN-γ by T helper cells/degradation rate of IFN-γ based on its half-life	$\frac{ng}{ml\times cell}$	[34]
$\frac{{\boldsymbol{\lambda}}_{\mathbf{1}}}{{\boldsymbol{\tau}}_{\mathbf{3}}}$	8.9382 × 10^−13^	The constant production rate of TGF-β by tumor cells/death rate of TGF-β based on its half-life	$\frac{ng}{ml\times cell}$	[34]

**Fig. 3:**
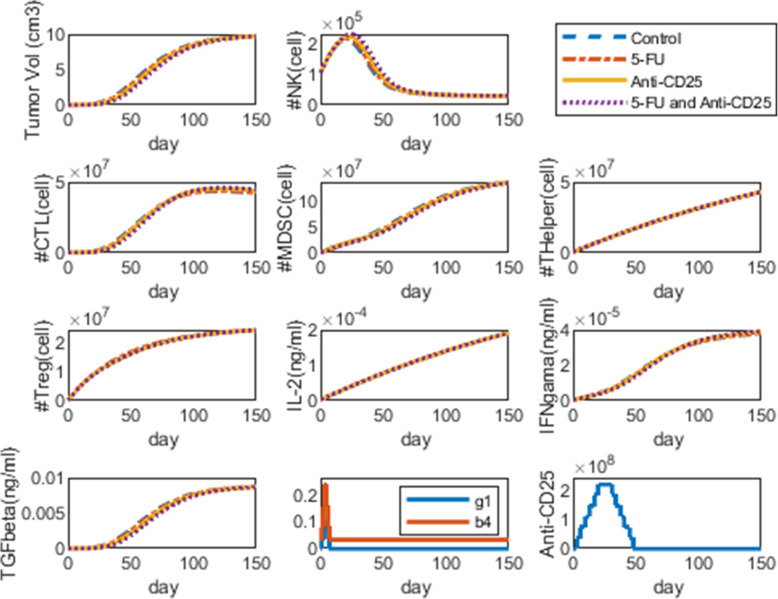
Dynamics of all species in strategies: control, 5-FU, Anti-CD25, and combination therapy. The blue dashed lines show predicted dynamics of cells/cytokines in the control group, and the red lines show with 5-FU treatment (on days 1, 2, 3, and 4 after tumor inoculation), the yellow lines show with anti-CD25 therapy (on days 3, 6, 10, 13, 17 and 20 after tumor inoculation) and purple lines depict the dynamics of cells/cytokines under combination therapy. In each subplot (except for the second and third figures in the last panel), the y-axis represents the number/concentration of cell population/cytokine, and the x-axis represents the time in days after tumor inoculation. The initial population/concentration of tumor cells, NKs, CTLs, MDSCs, TCD4+, Treg, IL-2, IFN-γ, and TGF-β have all been set to 6 × 10^5^, 105840, 21 × 10^4^, 3 × 10^3^, 564480, 42336, 2.5107 × 10^−6^, 3.9348 × 10^−7^, 5.363 × 10^−7^, respectively. The second and third figures in the fourth panel are parameters and terms related to 5-FU and anti-CD25 therapies, respectively

We demonstrated the cytotoxic effects of anti-CD25 and 5-FU therapies in a separate or in a combinatorial manner on the dynamics of pancreatic tumor cells. We considered the initial population of the tumor cells to be 6 × 10^5^, based on in vivo data of study [23], and the initial condition for NKs, CTLs, MDSCs, TCD4+ cells, and Treg cells to be 105840, 21 × 10^4^, 3 × 10^3^, 564480, 42336, respectively [34]. Also, the initial concentration of cytokines IL-2, IFN-γ, and TGF-β are 2.5107 × 10^−6^, 3.9348 × 10^−7^, 5.363 × 10^−7^, respectively, that are computed by eqs. 10–12. The dynamics of all cells/cytokines in different strategies including control, 5-FU, anti-CD25, and combination therapy are shown in different panels of Fig. 3. As shown in Fig. 3, injection of 5-FU on days 1, 2, 3, and 4 after tumor inoculation on day 0, and applying anti-CD25 therapy on days 3, 6, 10, 13, 17, and 20, affected dynamics of tumor cells and other factors of the model. To further investigate the effects of treatments on the dynamics of tumor cells, we computed the dynamics of inhibition percentage of instantaneous tumor size and average tumor size. Also, analysis of interaction among treatments was carried out to investigate the synergistic, additive, or antagonistic effects of treatments in a combinatorial manner. Finally, we optimized the timings of 5-FU and anti-CD25 injections by GA which results are provided in the next sections.

### Analysis of interactions among treatments

In this section, we aim to analyze the interaction among treatments. To determine synergistic, additive, or antagonistic effects of the combinatorial regimen of anti-CD25 and 5-FU therapies, we used the Bliss combination index (CI) [37]. We considered the instantaneous tumor size and the average tumor size as outcomes and we computed the CI values by setting different administration timings for anti-CD25 and 5-FU therapies. The interpretation of this analysis was based on the curve distance from the reference line and also the direction of the curve based on the reference line. For example, CI < 1 represents two treatments have synergistic effect if combined, CI = 1 indicates the additive effect of two treatment combination, and CI > 1 infers the antagonistic effect. The green dashed line indicates the threshold below which the 5-FU treatment and anti-CD25 treatment have a synergistic effect [37].

We defined the efficacy of anti-CD25 treatment (*ϕ*_*a*_) at time point *t* by *E*(*ϕ*_*a*_ = 1, *ϕ*_5_ = 0, t) and the efficacy of 5-FU treatment (*ϕ*_5_) at time point *t* by *E*(*ϕ*_*a*_ = 0, *ϕ*_5_ = 1, t) and their combination efficacy at time point *t* by *E*(*ϕ*_*a*_ = 1, *ϕ*_5_ = 1, *t*) that the formula of efficacy is as follows:

$$ E\left({\phi}_a=i,{\phi}_5=\mathrm{j},\mathrm{t}\right)=\frac{C\left({\phi}_a=0,\kern0.5em {\phi}_5=0,\mathrm{t}\right)-C\left({\phi}_a=i,\kern0.75em {\phi}_5=j,\kern0.75em \mathrm{t}\right)}{C\left({\phi}_a=0,\kern0.75em {\phi}_5=0,\kern0.75em \mathrm{t}\right)},i,j=0,1 $$

where *C*(*ϕ*_*a*_ = *i*,   *ϕ*_5_ = *j*,   t) represents the tumor cell population at day t in control case (*i = 0, j = 0*), in 5-FU treatment case (*i = 0, j = 1*), in anti-CD25 treatment case (*i = 1, j = 0*) and in combination therapy case (*i = 1, j =* 1). If the tumor population at day *t*, *C*(*ϕ*_*a*_ = *i*,   *ϕ*_5_ = *j*,   t), is smaller than the population in the control case, *C*(*ϕ*_*a*_ = 0,   *ϕ*_5_ = 0,   t), then the efficacy is a positive number and its value is between 0 and 100%.

**Fig. 4:**
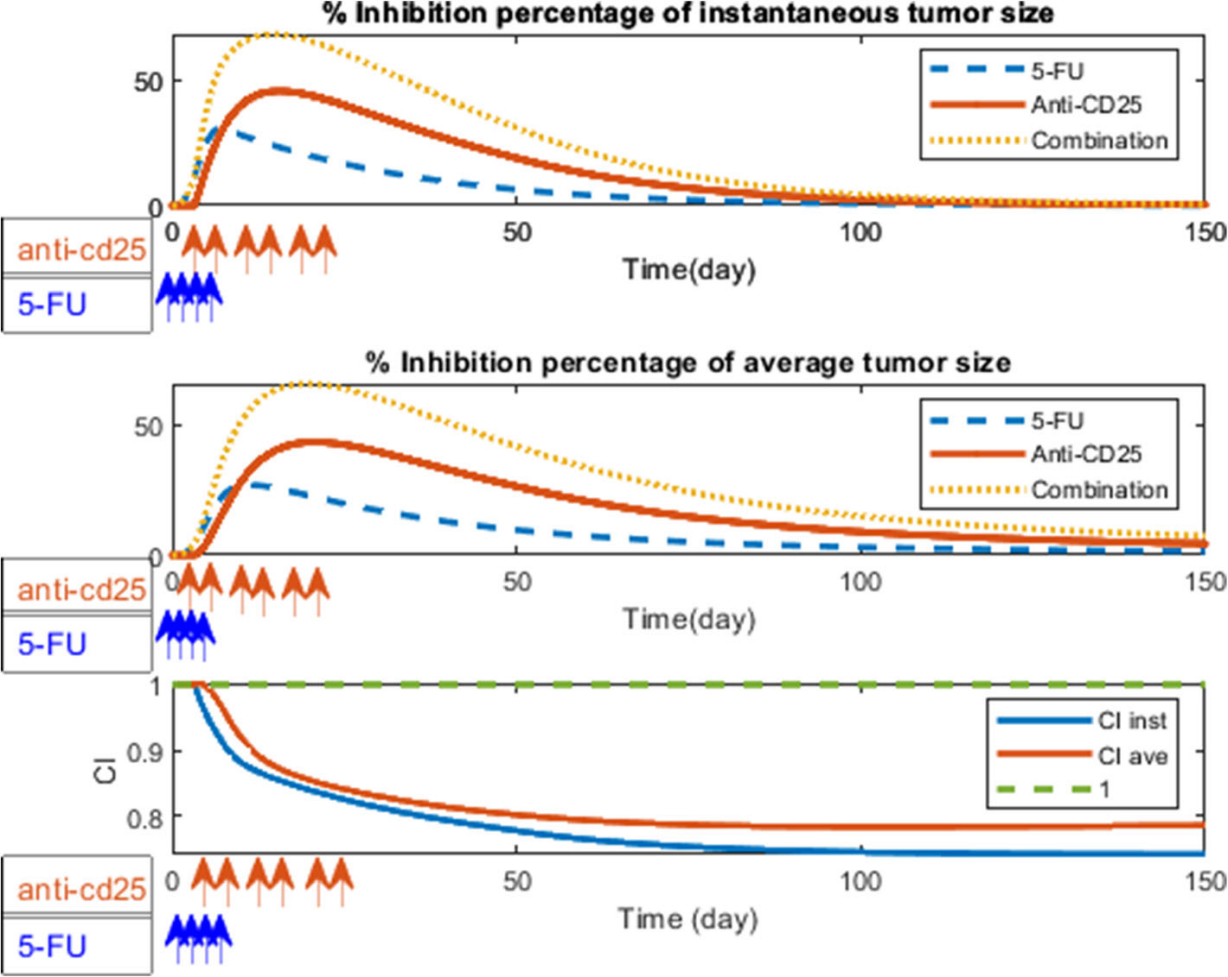
Comparison treatment efficacies and Analysis of interactions among treatments. The overall efficacy of 5-FU treatment, anti-CD25 treatment, and combination therapy was plotted as the percentage of tumor growth inhibition using instantaneous tumor size (first panel) and average tumor size (second panel) as outcomes. The blue line represents the inhibition percentage of tumor growth under 5-FU treatment on days 1, 2, 3, and 4 after tumor injection calculated based on the instantaneous tumor size during time and the red line under anti-CD25 treatment on days 3, 6, 10, 13, 17 and 20 after tumor inoculation and yellow line under combination therapy. (Third panel) The Bliss combination index (CI) for 5-FU and Anti-CD25treatments combination using instantaneous tumor size (*CI inst*) and average tumor size (*CI ave*) as an outcome. *CI < 1* represents the synergistic effect of two treatments, *CI = 1* additive, and *CI > 1* antagonistic effect. The green dash-line indicates the threshold below which the 5-FU treatment and anti-CD25 treatment have a synergistic effect

**Fig. 5:**
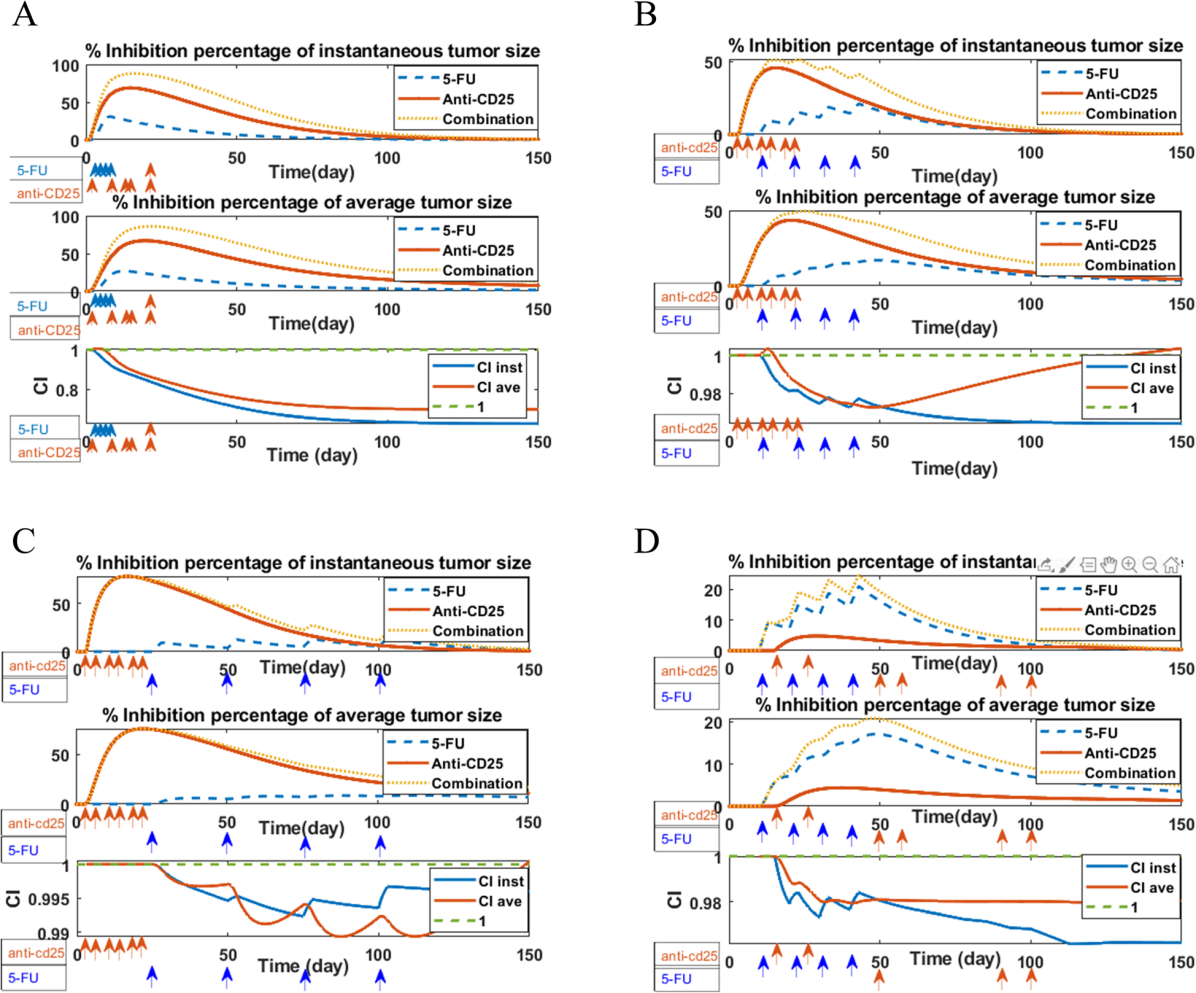
Comparison treatment efficacies and Analysis of interactions among treatments. The overall efficacy of 5-FU treatment, anti-CD25 treatment, and combination therapy was plotted as the percentage of tumor growth inhibition using instantaneous tumor size (first panel of Fig. 5. **A**, **B**, **C**, and **D**) and average tumor size (second panel of Figs. 5. **A**, **B**, **C**, and **D**) as outcomes. The blue lines represent the inhibition percentage of tumor growth under 5-FU treatment on a specified time setting in each subplot that is calculated based on the instantaneous tumor size during time and the red lines under anti-CD25 treatment and yellow lines under combination therapy. (Third panel of Fig. 5. **A**, **B**, **C**, and **D**) The Bliss combination index (CI) for 5-FU and Anti-CD25 treatments combination using instantaneous tumor size (*CI inst*) and average tumor size (*CI ave*) as outcome shows the synergistic interaction of treatments

As depicted in the first and second panels of Figs. 4 and 5. A, B, and C, we found although 5-FU therapy could suppress the tumor progression, anti-CD25 therapy has more regression impact than 5-FU therapy on the pancreatic tumor cell dynamics. Also, in silico assessments revealed the synergistic combinatorial manner has the most killing effect on tumor cells. This finding is consistent with in vivo data set that is used for model calibration (Fig. 2). The third panel of Figs. 4 and 5. A, shows a strong synergistic effect of the combination of 5-FU and anti-CD25 (in specified efficient design), while as depicted in the third panel of Figs. 5. B, C, and D, the ineffective administration of treatments (time settings) resulted in a poor synergy among treatments and consequently low efficacy in tumor degradation. Therefore, as shown in the third panel of Figs. 4 and 5. A, an appropriate schedule of 5-FU and anti-CD25 caused a strong synergistic effect of combined therapy on pancreatic tumor size and as time goes by, this effect becomes stronger (more distance from 1), while as shown in Figs. 5. B, C, and D, the specified timings of combination therapy led to an additive, an antagonistic, or poor synergy of treatments (lead to 1 or become > 1). Moreover, we realized that the combined therapy has a more synergistic effect on the instantaneous tumor size rather than averaged tumor size.

### Optimization of therapy

In this section, we aim to answer this question that what is the optimal combination and frequency of drug administration to present clinically significant conclusions? Dosing and timing of drug exposure determine the toxicity sustained in the tumor. Since the present mathematical model is configurable for 5-FU and anti-CD25 injection timing, we can optimize the timing of drug administration with GA to minimize the tumor burden. Since it has been shown that accumulation of pro-tumor immune cells such as Tregs and MDSCs mediate tumor cell regrowth, 18 to 20 days after tumor injection [48], we apply 5-FU and anti-CD25 therapies before day 20 to suppress the MDSCs and Tregs, respectively. To prevent toxic side effects of drugs, we assume that a maximum of two doses of each drug is allowed in each injection. The infusion times of 5-FU and anti-CD25 are denoted by ***t***_**5*F***_ and ***t***_***CD***_, respectively, that ${\boldsymbol{t}}_{\mathbf{5}\boldsymbol{F}}=\left[{t}_{5{F}_1},{t}_{5{F}_2},\dots, {t}_{5{F}_{N1}}\right]$**,** ${\boldsymbol{t}}_{\boldsymbol{CD}}=\left[{t}_{CD_1},{t}_{CD_2},\dots, {t}_{CD_{N2}}\right]$ and *N*1 (in the present study we assume 4), *N*2 (we assume 6) represent the preassigned number of times that 5-FU and anti-CD25 will be injected, respectively. GA estimate the optimal timings of 5-FU (***t***_**5*F***_^∗^) and anti-CD25 (***t***_***CD***_^∗^) by minimizing the following cost function to minimize the population of cancer cells in treatment groups:

$$ cost=\frac{1}{3N}\sum \limits_{i=1}^N{\left(\frac{C_{5F}\left({t}_i\right)}{C_{ctrl}\left({t}_i\right)}\right)}^2+{\left(\frac{C_{CD}\left({t}_i\right)}{C_{ctrl}\left({t}_i\right)}\right)}^2+{\left(\frac{C_{comb}\left({t}_i\right)}{C_{ctrl}\left({t}_i\right)}\right)}^2 $$

$$ \left[{C_{5F}}^{\ast}\left({{\boldsymbol{t}}_{\mathbf{5}\boldsymbol{F}}}^{\ast}\right),{C_{CD}}^{\ast}\left({{\boldsymbol{t}}_{\boldsymbol{CD}}}^{\ast}\right),{C_{comb}}^{\ast}\left({{\boldsymbol{t}}_{\mathbf{5}\boldsymbol{F}}}^{\ast },{{\boldsymbol{t}}_{\boldsymbol{CD}}}^{\ast}\right)\kern0.5em \right]=\underset{{{\boldsymbol{t}}_{\mathbf{5}\boldsymbol{F}}}^{\ast },{{\boldsymbol{t}}_{\boldsymbol{CD}}}^{\ast }}{\min }(cost) $$

which *C*_*ctrl*_(*t*_*i*_), *C*_5*F*_(*t*_*i*_), *C*_*CD*_(*t*_*i*_), and *C*_*comb*_(*t*_*i*_) represent the population of cancer cells at time point *t*_*i*_, *i* = 1, 2, …, *N* in control group, 5-FU group, anti-CD25 group, and combination therapy group, respectively. Also, *t*_*N*_ is the end time of simulation which is day 100. The quantities *C*_5*F*_^∗^(***t***_**5*F***_^∗^), *C*_*CD*_^∗^(***t***_***CD***_^∗^), *C*_*comb*_^∗^(***t***_**5*F***_^∗^, ***t***_***CD***_^∗^) represent the minimized tumor population by applying 5-FU, anti-CD25, and combination therapy in optimal times, respectively.

**Fig. 6:**
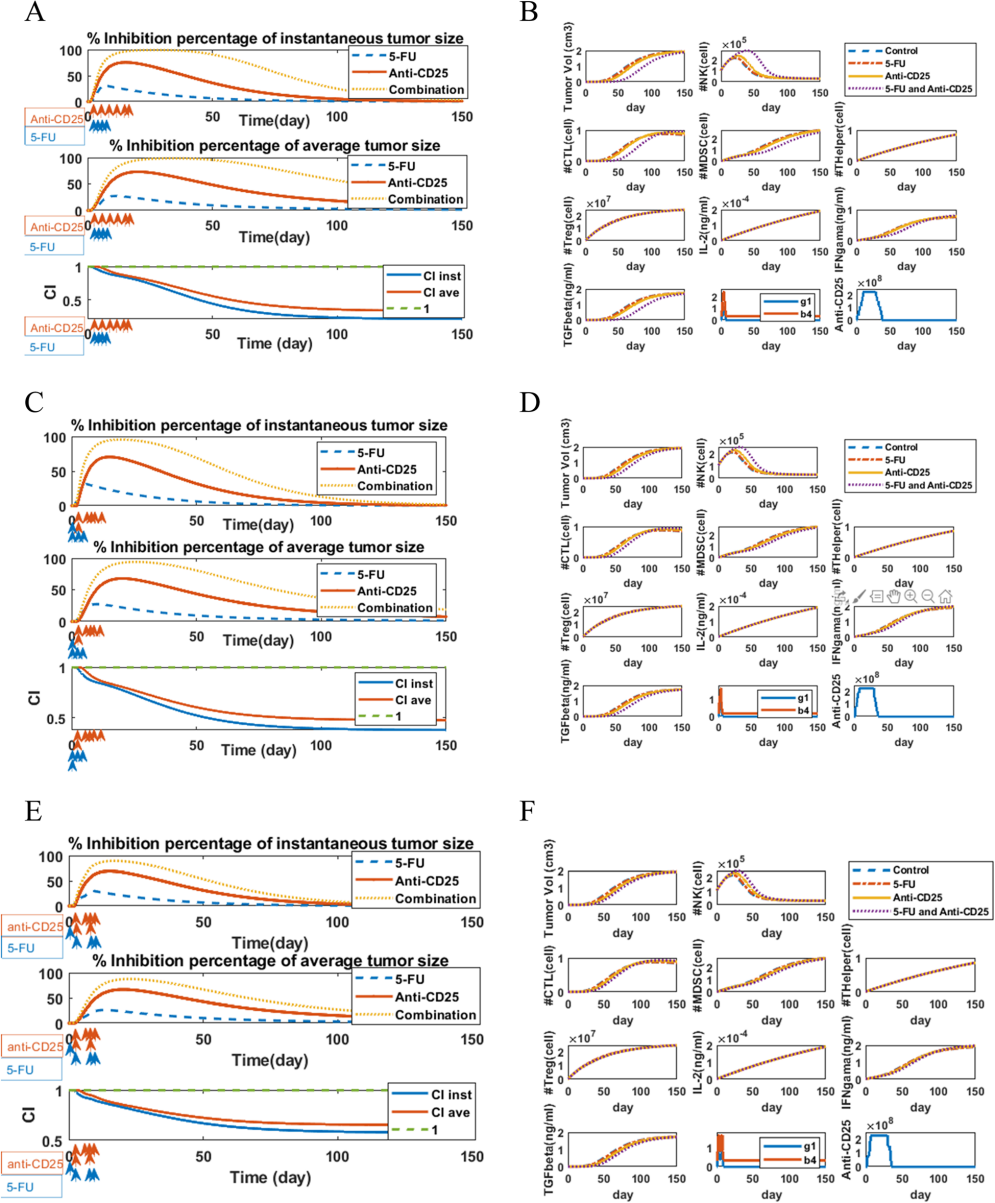
Dynamics of all cells/cytokines in strategies: control, 5-FU, Anti-CD25, and combination therapy with an optimized treatment schedule and comparison treatment efficacies and analysis of interactions among treatments. The blue lines in Fig. 6. **B**, **D**, and **F** show predicted dynamics of cells/cytokines in the control group, and the red lines show with 5-FU treatment (on specified time points), the yellow lines show with anti-CD25 therapy (on specified time points) and purple lines depict the dynamics under combinatorial manner. In each subplot in Fig. 6. **B**, **D**, and **F** (except for the second and third figures in the last panel), the y-axis represents the number/concentration of cell population/cytokine, and the x-axis represents the time in days after tumor inoculation. The initial condition is the same as those given in Fig. 3. The second and third figures in the fourth panels of fig. 6. **B**, **D**, and **F** show the terms related to 5-FU and anti-CD25 therapies, respectively. The overall efficacy of 5-FU treatment, anti-CD25 treatment, and combination therapy was plotted as the percentage of tumor growth inhibition using instantaneous tumor size (first panel of figs. 6. **A**, **C**, and **E**) and average tumor size (second panel of figs. 6. **A**, **C** and **E**) as outcomes. The blue line represents the inhibition percentage of tumor growth under 5-FU treatment on specified time points after tumor injection calculated based on the instantaneous tumor size during time and the red line under anti-CD25 treatment on specified time points after tumor inoculation and the yellow line under combination therapy. (Third panel of fig. 6. **A**, **C** and **E**) The Bliss combination index (CI) for 5-FU and Anti- treatments combination using instantaneous tumor size (*CI inst*) and average tumor size (*CI ave*) as an outcome

As shown in first and second panels of Figs. 6. A, 6.C and 6.E, optimization of 5-FU anti-CD25 therapies caused the tumor inhibition percentage in combination therapy regimen to reach its maximum value (100%). As shown in third panel of Figs. 6. A, B and C, optimization of timing of 5-FU and anti-CD25 treatments caused these treatments to have a strong synergistic effect.

### Results of fuzzy analysis

**Fig. 7:**
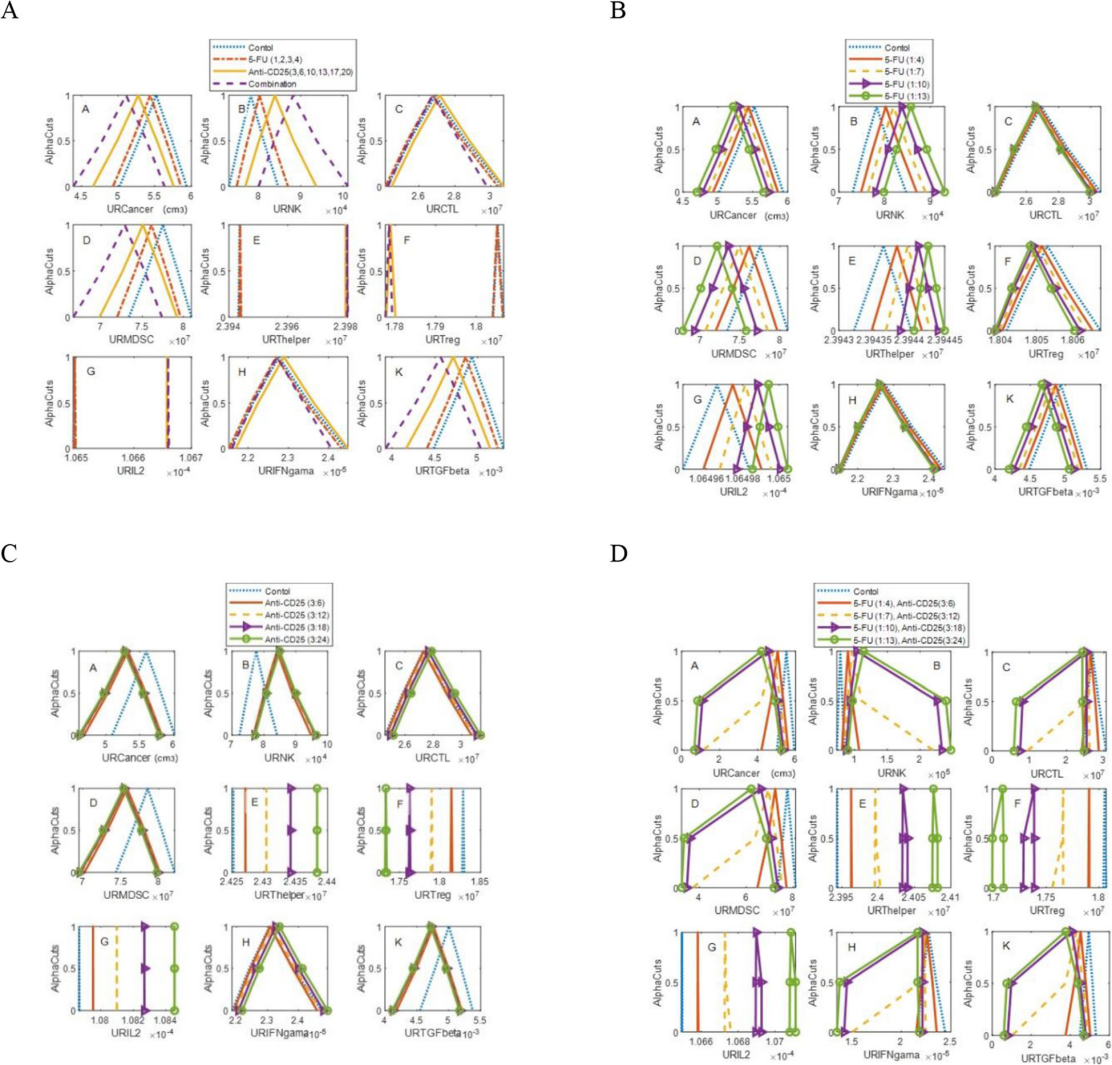
In silico assessment of treatments in the fuzzy setting. The membership function of the average of dynamics of cancer cells (panel **A** of figs. 7. A, B, C, and D), NK cells (panel **B** of figs. 7. A, B, C, and D), CTLs (panel **C** of figs. 7. A, B, C, and D), MDSCs (panel **D** of figs. 7. A, B, C, and D), T helper (panel E of figs. 7. A, B, C, and D), Treg (panel **F** of figs. 7. A, B, C, and D), cytokine IL-2 (panel **G** of figs. 7. A, B, C, and D), IFN-γ (panel **H** of figs. 7. A, B, C, and D), and TGF-β (panel **K** of figs. 7. A, B, C, and D) in the time interval from the start of therapies to day 150 for injection of 5-FU (on days 1, 2, 3, and 4) and anti-CD25 (on days 3, 6, 10, 13, 17 and 20) in fig. 7**A**, the different timing of 5-FU injection (fig. 7**B**), and for different timing of anti-CD25 injection (fig. 7**C**), and different timings of the combination of 5-FU and anti-CD25 (fig. 7**D**), in the fuzzy setting of kinetic parameter *a*_1_ = (**0.9**, **1**, **1.1**) **× 4.3992** ***×*** **10**^***−*2**^

Due to the parametric uncertainty in the model, it seems that the evaluation of treatments in the fuzzy setting is more appropriate than the crisp setting. In this section, we aim to predict uncertain dynamics of cells/cytokines of the TIS model affected by the uncertainty of parameters. We assign a triangular membership function to some kinetic parameters and investigate the effect of that parametric uncertainty on the dynamics of model constituents. The simulation of parametric uncertainty with a fuzzy theorem in an ODE model of this study is similar to that used in other models including stochastic Petri net and continuous Petri net [43, 45]. We simulated the ODE model with fuzzy parameters and in the presence and absence of 5-FU and anti-CD25 therapies. In silico assessment of model in fuzzy setting revealed that 5-FU therapy on days 1, 2, 3, and 4 and anti-CD25 therapy on days 3, 6, 10, 13, 17, and 20 and combinatorial manner caused the uncertainty band of cancer cells (panel A of Fig. 7. A), MDSCs (panel D of Fig. 7. A), and Tregs (panel F of Fig. 7. A), shift left, toward lower population (volume) of these cells and the uncertainty band of NK cells (panel B of Fig. 7. A), and T helper cells (panel E of Fig. 7. A), shift right, toward an upper population of these cells.

The results of increasing the frequency of 5-FU injections are depicted in Fig. 7. B. By increasing the frequency of 5-FU injections, the uncertainty band of cancer cells (panel A of Fig. 7. B), MDSCs (panel D of Fig. 7. B), and Tregs (panel F of Fig. 7. B) shift left toward the lower population of these cells, respectively while the uncertainty band of NK cells (panel B of Fig. 7. B) and T helper cells (panel E of Fig. 7. B) shift right toward the upper population of these cells. As shown in Fig. 7. C, the anti-CD25 treatment caused the uncertainty band of cancer cells (panel A of Fig. 7. C), the MDSCs (panel D of Fig. 7. C), and Tregs (panel F of Fig. 7. C) for no treatment case to be shifted left toward the lower population of these cells and the uncertainty band of NK cells to be shifted right toward the upper population of NK cells, but by increasing frequency of anti-CD25 treatment, the uncertainty band of cancer cells, NK cells, and MDSCs cells does not move effectively. On the other hand, by increasing the frequency of anti-CD25 treatments, the uncertainty band of T helper (panel F of Fig. 7. C) and Tregs (panel E of Fig. 7. C) shift right and left, respectively. Finally, as depicted in Fig. 7. D, increasing the regimens of both 5-FU and anti-CD25 in a combinatorial manner caused the uncertainty band of cancer cells (panel A of Fig. 7. D), MDSCs (panel D of Fig. 7. D), and Tregs (panel F of Fig. 7. D) to be shifted left toward the lower population of these cells while caused the uncertainty band of NK cells (panel B of Fig. 7. D) and T helper cells (panel E of Fig. 7. D) to be shifted right toward the higher population of these cells.

### Results of global sensitivity analysis

**Fig. 8:**
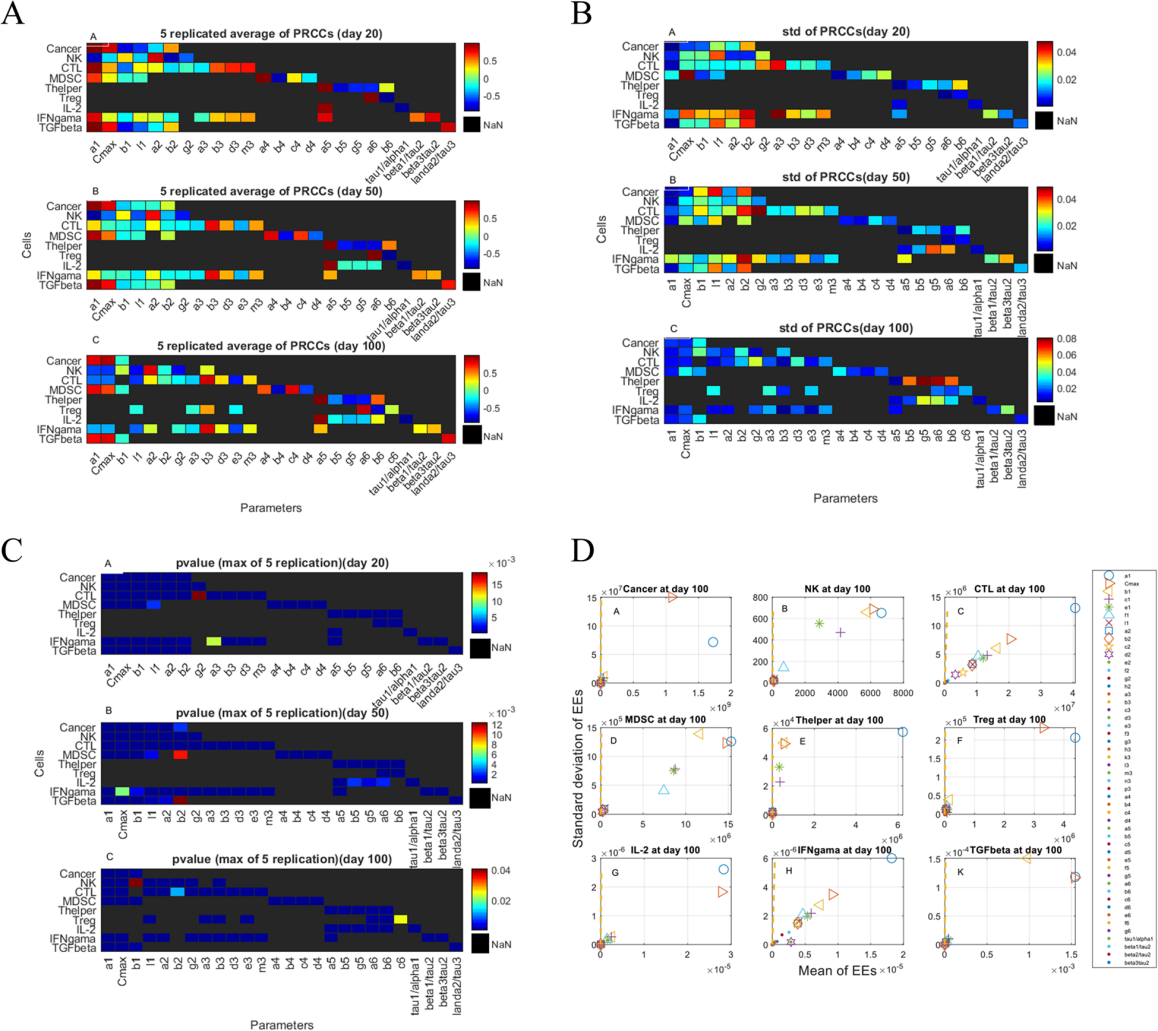
GSA analysis. Statistically significant PRCC values (*p*-value< 0.05) for tumor cells, NK cells, CTLs, MDSCs, T helper cells, Tregs, IL-2, IFN-γ, and TGF-β at days 20 (A. A), 50 (A. B) and 100 (A. C) after tumor injection. The mean of PRCC values for five replications of PRCC analysis was depicted in each pixel. Black pixels (‘NaN’) show ‘not a number’ and represent no significant correlation between outcome measures (population/concentration of cells/cytokines, elements in the vertical axis) and kinetic parameters of the model (elements in the horizontal axis). The standard deviation of significant PRCC values (*p*-value< 0.05) for five replications of PRCC analysis for tumor cells, NK cells, CTLs, MDSCs, T helper cells, Tregs, IL-2, IFN-γ and TGF-β at days 20 (B. A), 50 (B. B) and 100 (B. C) after tumor injection. The standard deviation of significant PRCC values for five replications of PRCC analysis was depicted in each pixel. *P*-values of PRCC analysis for tumor cells, NK cells, CTLs, MDSCs, T helper cells, Tregs, IL-2, IFN-γ, and TGF-β at days 20 (C. A), 50 (C. B), and 100 (C. C) after tumor injection. The maximum of *p*-values for five replications of PRCC analysis was depicted in each pixel. (D) The absolute mean value and standard deviation of the elementary effects test. Figures 8. D presents the relative importance of kinetic parameters of the TIS model, considering the population/concentration of cells/ cytokines at day 100 as the read-out, including the population of tumor cells (D. A), NK cells (D. B), CTLs (D. C), MDSCs (D. D), T helper cells (D. E), Tregs (D. F), IL-2 (D. G), IFN-γ (D. H) and TGF-β (D. K). Each kinetic parameter is specified by two Morris indices, *μ* *(horizontal axis) and *σ* (vertical axis), which describe the significance of the effects and the interaction effects, respectively

We carried out global sensitivity analysis (GSA) regarding the population/concentration of cells/cytokines at days 20, 50, and 100 in the no treatment case as well as all kinetic parameters of the system described in Table 1. By using the GSA method in study [46], we carried out Latin hypercube sampling (LHS) and produced 10,000 samples to compute the partial rank correlation coefficient (PRCC) and the *p*-values regarding the population/concentration of cells/cytokines at days 20, 50 and 100 after tumor inoculation. In LHS, we took the range of parameters ½ to twice their values in Table 1. We performed the PRCC analysis five times and calculated the mean (Fig. 8a) and standard deviation (Fig. 8b) of the statistically significant correlation values (*p*-value< 0.05). The computed p-values (Fig. 8c) are the maximum values between five times replications. In Fig. 8a, b and c, only the significant correlation values (*p*-value< 0.05) are reported. As shown in panel A of Fig. 8. A, we see that the population of tumor cells at day 20 after tumor injection is positively correlated to the pro-tumor parameters *a*_1_ (tumor growth rate), *C*_*max*_ (maximum tumor size), and anti-immune parameter *b*_2_ (death rate of NK cells) while it is negatively correlated to the anti-tumor parameters *b*_1_ (NK-mediated killing rate of tumor cells), *l*_1_ (depth of access of immune cell to tumor cells) and *a*_2_ (the constant source of NK cells). In this instance, the negative correlation between tumor population at day 20 and parameter *l*_1_ represents that if parameter *l*_1_ is increased, the depth of immune cells access to tumor cells will increase, resulting in the killing of more tumor cells and consequently a decrease in the tumor cell population. Also, we see that the pro-tumor parameters, namely, *a*_1_, *C*_*max*_, and *b*_2_ are negatively correlated to the NK cell population at day 20 while anti-tumor parameters such as *b*_1_ and *a*_2_ are positively correlated to the NK cell population at day 20. The NK cell population at day 20 is negatively correlated to the *l*_1_ (depth of access of immune cells to the tumor) and *g*_2_ (inactivation rate of NKs by tumor cells). Since there is a strong correlation between parameter *b*_3_ (Maximum tumor-mediated CTL recruitment rate) and the CTL population at day 20, and according to the second term of eq. (15) that describe CTL recruitment, the CTL population is strongly affected by the tumor cell population (as the tumor cell population increases/decreases, the immune cell population increases/decreases). Since both tumor cells and CTLs have similar dynamics, their populations at day 20 are positively correlated with parameters *a*_1_ and *C*_*max*_. According to panel A of Fig. 8. A, there exist a negative correlation between *b*_1_ (maximum killing rate of tumor cells by NKs), *b*_2_ (death rate of NKs), *g*_2_ (maximum inactivation rate of NKs by NK-tumor interactions), and CTL population at day 20. The other correlation values are depicted in panels A, B, and C of Fig. 8. A, are similarly interpretable.

Morris GSA was used to identify which of 52 kinetic parameters of the model have a significant effect on cell dynamics. For 52 kinetic parameters (with 10% perturbations), EE test using Morris sampling strategy were taken into account by setting 10 levels in the sampling grid and 1000 trajectories to compute the mean μ^∗^ and standard deviation *σ*. The identified most influential kinetic parameters with respect to the mean μ^∗^ and interaction effect *σ* are depicted in Fig. 8d. The parameters with large μ^∗^ values indicate the linear or additive effects while the parameters with large *σ* values indicate interaction effects. The dashed line ${\upmu}^{\ast }=\frac{2\sigma }{sqrt(r)}$ (r is the number of trajectories) which all parameters are below that, translates into 95% confidence interval. Morris analysis was performed by considering the population of tumor cells, NK cells, CTLs, MDSCs, T helper, Tregs, and cytokines IL-2, IFN-γ, and TGF-β (in no treatment case) at day 100 of simulation as a read-out.

Our data revealed that the parameters *a*_1_, *Cmax*, reflecting the tumor growth rate and carrying capacity of tumor cells, respectively, are the most influential parameters for the tumor cell output (panel A of Fig. 8. D). The parameter *a*_1_ has the most linear effect while the parameter *Cmax* has the most interaction effect on the dynamics of tumor cells. As depicted in panel B of Fig. 8. D, the parameters *a*_1_, *Cmax*, *b*_1_ (NK-mediated tumor cell killing rate), *c*_1_ (CTL-mediated tumor cell killing rate), *e*_1_ (Rate of the suppressive effect of Treg on CTL-mediated tumor cell killing) were predicted to play an important role in NK cells dynamics. The parameters *a*_1_, *Cmax*, *b*_1_ have both linear and interaction effects while the parameters *c*_1_, *e*_1_ have interaction effects on the dynamics of NK cells. Also parameter *a*_1_ has strong interaction and liner effects on CTL’s dynamics (panel C of Fig. 8. D). the results of the EE test show that parameters *a*_1_, *Cmax* have linear effects while parameter *b*_1_ has an interaction effect on the dynamics of MDSCs (panel D of Fig. 8. D). Also, parameters *Cmax*, *b*_1_ have an interaction effect, and parameter *a*_1_ has both linear and interaction effects on the dynamics of T helper cells (panel E of Fig. 8. D). As depicted in panel F of Fig. 8. D, the parameters *Cmax* and *a*_1_ have interaction and both linear and interaction effects, respectively. As shown in panels F, G, and H of Fig. 8. D, the parameter *a*_1_ has most both linear and interaction effects on dynamics of Tregs, IL-2, and IFN-γ while the parameter *Cmax* has both interaction and linear effects on dynamics of Tregs and IL-2, respectively. As shown in panel K of Fig. 8. D, the parameters *a*_1_, *Cmax* have linear effects and parameter *b*_1_ has an interaction effect on the dynamics of TGF-β.

## Discussion

Mathematical modeling of complex networks of tumor-immune system (TIS) interactions is constantly advancing. Mathematical oncology helps us to better understand cancer biology, to assess the efficacy and toxicity of different treatment planning, and to predict dynamics of cancer and immune system behaviors during treatment. Systematic analysis of TIS by sophisticated modeling approaches can be used to refine and optimize drug dosing and scheduling. Dedicated modeling of chemotherapy/immunotherapy drug regimens by pharmacokinetic/pharmacodynamics models in oncology should result in the improved clinical efficacy of therapy and decreased toxicity. Mathematical modeling provides a personalized medicine approach to design a better efficacy–toxicity balance of therapy.

**Fig. 9:**
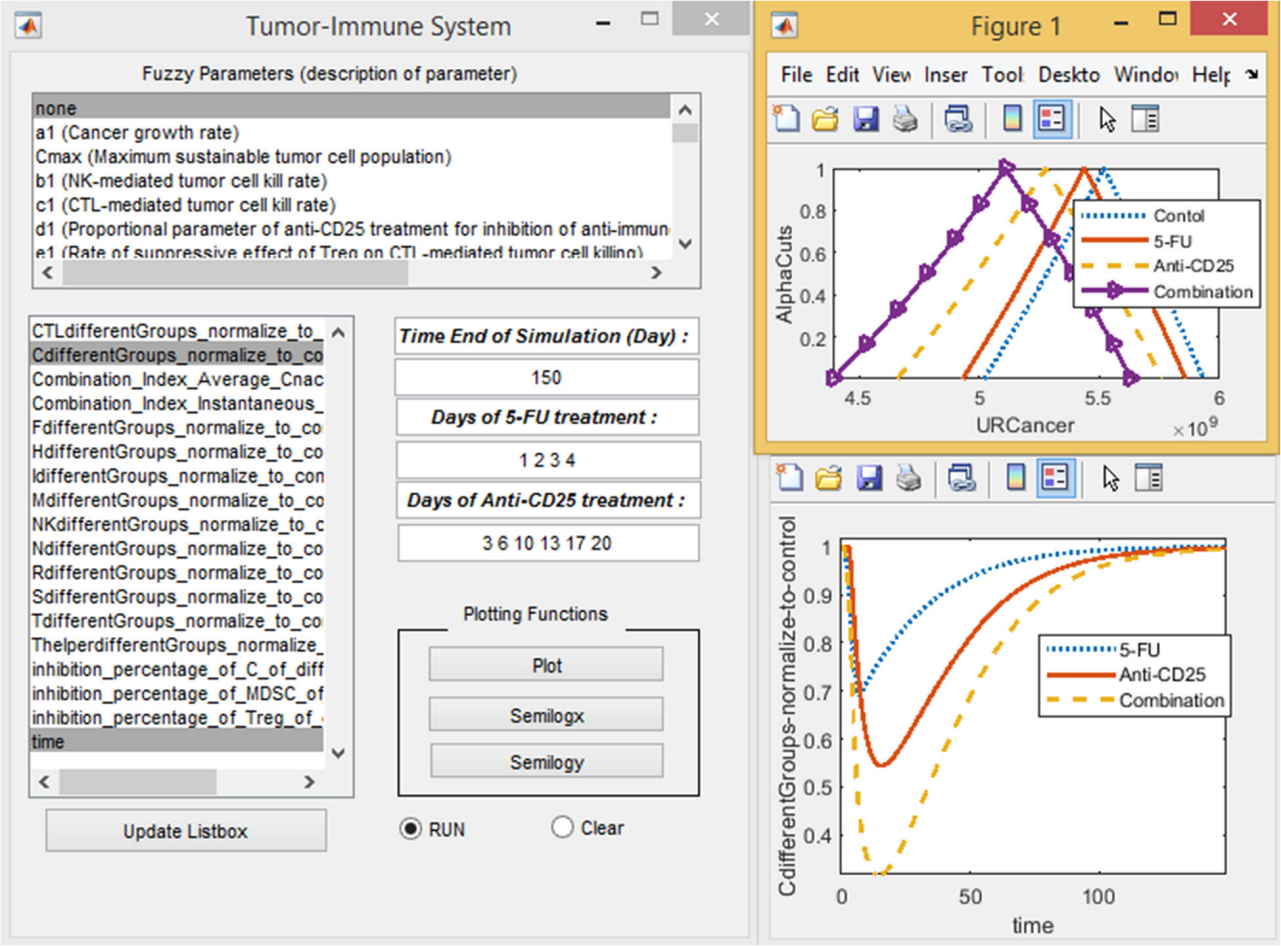
The Graphical user interface (GUI) of the model. The user-friendly GUI of TIS with regarding fuzzy/crisp kinetic parameters for in silico assessment of 5-FU and anti-CD25 therapies

PDAC creates a tumor microenvironment that enhances tumor progression and metastasis. Tumor-induced dysfunction of immune cells is a critical issue in this microenvironment. Tumor cells induce immune suppression by many different mechanisms, including accumulation of regulatory T cells (Treg) and myeloid-derived suppressor cells (MDSCs). Interactions of immune system constituents including pro/anti-tumor cells and cytokines with tumor cells create a complex network with unknown behaviors that can be predicted by mathematical modeling. Mathematical modeling of the mutual interactions of tumor cells with the immune system in the tumor microenvironment helps us to understand the various processes involved in tumor growth and metastasis. The advent of mathematical modeling can help us to address treatment scheduling while simultaneously helping to unravel the processes driving therapeutic responses. The understanding complex interactions of tumor-immune system agents will elucidate the mechanisms of action of chemotherapy and immunotherapy drugs and lead to modify treatment schedules. Theoretically, a combined anti-CD25 immunotherapy and 5-FU chemotherapy would elicit a greater immune response. However, regimen scheduling of combination 5-FU and anti-CD25 therapies has yet to be established. Here, we calibrate a mathematical model of the tumor-immune system (TIS) to in vivo data from 5-FU and anti-CD25 therapies to simulate the complex interplay between the invading tumor cells and innate and adaptive immune system constituents. The model helps us to implement an in silico clinical trial to test combination 5-FU and anti-CD25 therapies and optimize combination regimens to improve treatment efficacy. For this aim, we designed a user-friendly graphical user interface (GUI) unit (Fig. 9) that is configurable for 5-FU and anti-CD25 treatment timing in both crisp/fuzzy settings. The present GUI, as a rigorous simulation framework, help us to predict dynamics of TIS constituents (cell/ cytokine) in different schedules of 5-FU and anti-CD25 therapies or absence of treatment with (fuzzy setting) and without (crisp setting) regarding parametric uncertainty (MATLAB codes of GUI and the additional file are in supplementary file).

In a study, *Peng* et al. designed a mathematical model to evaluate the efficacy of different treatments for prostate cancer and measured the interactions between different treatments based on tumor size at a given time point [37]. In the present study, we evaluated the effect of 5–FU and anti-CD25 treatments for pancreatic cancer by an in-vivo parameterized mathematical model and calculated the dynamics of interactions between these treatments based on instantaneous and average tumor size over time. Using the present model, we can capture the dynamics of TIS constituents and investigate the effect of different treatment schedules on the dynamics of TIS agents. Also, *Peng* et al analyzed the effect of parameter perturbations on cell dynamics by local sensitivity analysis methods while in the present study, we performed PRCC and EE tests which are global sensitivity analysis methods. Also, in the present study, we used fuzzy theorem to assess the effect of fuzzy uncertainty of kinetic parameters on dynamics of cells/ cytokines in the presence and absence of treatment modalities. The present model can be used as a rigorous simulation framework to predict the dynamics of TIS interactions and identify different behaviors of TIS in response to treatments. The kinetic parameters of the present model are estimated by GA and based on published data from 5-FU and anti-CD25 pancreatic cancer treatments. The model of the present study is formulated by ordinary differential equations that by deterministic rules simulate the complex behaviors and interactions of TIS agents. Although deterministic models cannot capture uncertain cell-to-cell interactions and stochastic behaviors of TIS agents and also intrinsic noise in signaling pathways that control different behaviors of TIS agents, they have been widely used to simulate complex interplay between the invading tumor cells, innate and adaptive immune system constituents to predict dynamics of TIS agents with or without different treatment modalities. In the present study, we simulated the effect of uncertainty of model kinetic parameters through fuzzy theorem and we assessed the effect of different 5-FU and anti-CD25 treatment schedules in both crisp and fuzzy settings of kinetic parameters. Fuzzification of model parameters can help us to capture fuzzy uncertainty of model parameters which is due to imprecise, missing, or incomplete data.

Although no experimental or clinical study was conducted on the combination therapy of 5-FU and anti-CD25 so far, we evaluate the crosstalk between the immune system components on which these therapies mediated their consequences in the tumor microenvironment. Recently, Siret C et al. unraveled the underlying interaction between immune system cells within the PDAC microenvironment with a focus on Treg cells and MDSCs. They demonstrated that in the PDAC microenvironment, MDSCs suppress CTL proliferation, induce CTL death and produce arginase 1 and reactive oxygen species (ROS) and simultaneously Treg cells inhibit the proliferation of T helper cells that all these consequences provided strong immunosuppression in the PDAC microenvironment. Also, they showed in-vivo depletion of MDSCs inhibits induction and recruitment of Treg cells in PDAC microenvironment and also ex-vivo co-culture assays of Treg cells and MDSCs revealed tumoral MDSCs induce the development and proliferation of Treg cells mediated by cell-to-cell crosstalk and conversely the presence of Treg cells leads to survival and increase of tumoral MDSCs [48]. Moreover, the positive direct interactions between MDSCs and Treg cells in the tumor microenvironment were shown in other cancers [49–53]. In melanoma, Treg cells induce tumoral MDSCs differentiation through the expression of B7H1 and also the expression of Indoleamine 2,3-dioxygenase (IDO) by tumor cells in a Treg cells dependent manner recruits and activate MDSCs in the tumor microenvironment [50–54]. Moreover, Re GL et al. surveyed the combination therapy of Cyclophosphamide, 5-FU, and IL-2 for solid tumors, and as we know IL-2 against anti-CD-25 therapy induces Treg cell proliferation. They reported the response duration of this combination therapy for pancreatic cancer was over 18 months and during this period, intravenous IL-2 in compared to subcutaneous administration leads to more platelet decrease, less platelet/lymphocyte decrease, and less Treg cells increase. However, the total number of lymphocytes and Treg cells increased after therapy [55].

## Conclusion

We integrate a mathematical model of 5-FU and anti-CD25 into a simulation framework to optimize their administration in combination therapy. Using this framework, we inferred a combination schedule for the treatment of PDAC that significantly improved treatment outcomes when compared to 5-FU and anti-CD25 separately and provided a standard combination regimen. Our findings outline a rational approach to therapy optimization with meaningful consequences for how we effectively design treatment schedules to maximize their success, and how we treat PDAC with combined 5-FU and anti-CD25 therapy. In silico assessment of the model reveals that the combination of 5-FU and anti-CD25 treatments has potentially improved therapeutic effects through preventing tumor-induced immune suppressive mechanisms within the PDAC microenvironment.

## Supplementary Information

**Additional file 1.**

## Data Availability

All data generated or analysed during this study are included in this manuscript and its supplementary information files which includes MATLAB code (Please open MATLAB codes by MATLAB software or see the MATLAB codes in the supplementary file) and description of GUI.

## Abbreviations

PDAC: Pancreatic ductal adenocarcinoma
Treg: Regulatory T cell
MDSC: Myeloid-derived suppressor cell
5-FU: 5-fluorouracil
CTL: Cytotoxic T lymphocyte
NK: Natural killer
IL-2: Interleukin-2
IFN-γ: Interferon-γ
TGF-β: Transforming growth factor-β
TIS: Tumor-immune system
GUI: Graphical user interface
ODE: Ordinary differential equation
NRMSE: Normalized root-mean square error
PRCC: Partial rank correlation coefficient
EE: Elementary effect
CI: Combination index
