# Supplementary material for: Prediction of anti-CD25 and 5-FU treatments efficacy for pancreatic cancer using a mathematical model
Source: BMC Cancer. 2021 Nov 15;21:1226. doi: 10.1186/s12885-021-08770-z (PMC8594222; doi:10.1186/s12885-021-08770-z)
Supplement: Supplementary file 1 — Additional file 1. [file 12885_2021_8770_MOESM1_ESM.docx]

**The supplementary file includes 1- title page and 2- description of Graphical User Interface (GUI) of the developed mathematical model (In this section we provided some screenshots of our GUI, please open the MATLAB codes provided in supplementary files by MATLAB software) and 3- MATLAB codes that are readable by MATLAB software.**

1. **Title page:**

**Title: Prediction of anti-CD25 and 5-FU treatments efficacy for pancreatic cancer using a mathematical model**

Sajad. Shafiekhani^1, 2, 3^, Hojat. Dehghanbanadaki^3, 4^, Azam. S. Fatemi^1, 2^, Jamshid. Hajati^5^, Amir. H. Jafari^1, 2*^

^1^Departments of Biomedical Engineering, School of Medicine, Tehran University of Medical Sciences.

^2^Research Center for Biomedical Technologies and Robotics, Tehran, Iran.

^3^Students’ Scientific Research Center, Tehran University of Medical Sciences, Tehran, Iran.

^4^ Metabolic Disorders Research Center, Endocrinology and Metabolism Molecular-Cellular Sciences Institute, Tehran University of Medical Sciences, Tehran, Iran

^5^ Departments of Medical Immunology, School of Medicine, Tehran University of Medical Sciences, Tehran, Iran.

**Running title:** Anti-CD25 & 5-FU combination efficacy for pancreatic cancer

^*^Corresponding author:

Amir. H. Jafari, Departments of Biomedical Engineering, School of Medicine, Tehran University of Medical Sciences, Tehran, Iran. Email: [h_jafari@tums.ac.ir](mailto:h_jafari@tums.ac.ir), Tel: +989122384410, Postal code: 1417613151

1. **A Graphical User Interface (GUI) of Tumor-Immune system (TIS) in MATLAB:**

**Appendix file for deciphering TIS interactions and for in silico assessment of 5-FU and anti-Treg therapies with fuzzy and crisp kinetic parameters via the developed GUI in MATLAB.**

Interplaying tumor cells with immune system constituents create a complex system that its dynamics can be predicted via mathematical modeling approaches. For this aim and for in silico assessment of anti-tumor therapies, we designed an ordinary differential equation model and parameterized it with in vivo data. The mathematical model of this study is configurable for injection timings of 5-FU and anti-Treg therapies. Due to imprecise, incomplete, or missing experimental data, the kinetic parameters of models are uncertain. In this study, we assigned fuzzy uncertain numbers instead of crisp values for kinetic parameters to capture this natural uncertainty. Therefore, in addition to analysis of TIS in the crisp setting, there is the ability to analyze the TIS in the fuzzy setting to capture the dynamics of the uncertainty band of cells/cytokines. In this section, we present the graphical user interface (GUI) designed in MATLAB for easy use of the model to decipher TIS interactions and assess the 5-FU and anti-Treg therapies in silico with fuzzy and crisp kinetic parameters.

We can adjust model parameters and inputs and then evaluate the TIS in different conditions to deepen our understanding of the tumor and immune system mechanisms. In this section, we show how to set up the model inputs step by step to test different immunological hypotheses in a simulation environment. Here we provide a collection of MATLAB screenshots outlining the workflow of simulating TIS regarding fuzzy/crisp kinetic parameters to evaluate the different regimens of 5-FU and anti-Treg treatments.


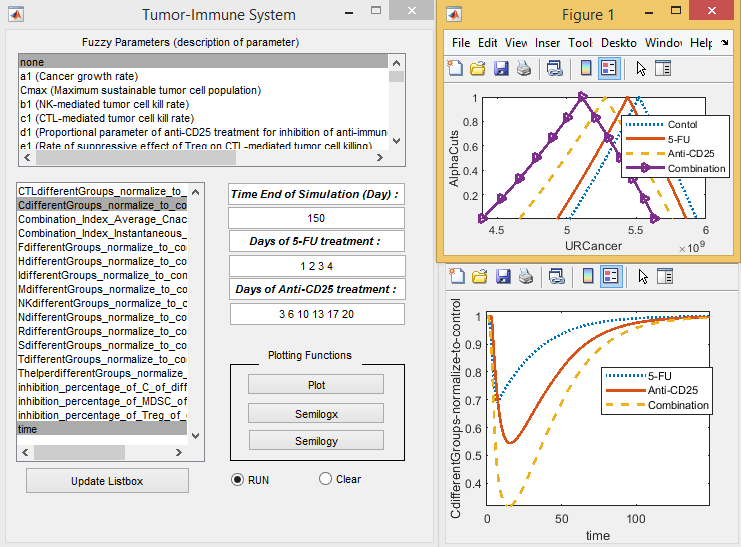


Figure 1. Graphical user interface (GUI) of TIS regarding fuzzy/crisp kinetic parameters for in silico assessment of 5-FU and anti-CD25 therapies.

As depicted on the left side of Figure 1, the GUI of TIS contains a list box for selecting fuzzy parameters along with a panel for adjusting the timing of model simulation and injection times of drugs and also a panel for showing the results of model simulation.


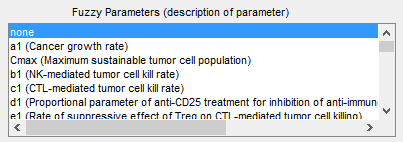


Figure 2. List box of fuzzy parameters and biological description of kinetic parameters. If select ‘none’, all parameters will be crisp.

This List box contains all the kinetic parameters of the TIS model. If we select 'none' it means that all kinetic parameters are crisp and there is no uncertainty in the kinetic parameters. By selecting one or multiple parameters in the list box, the program assigns a triangular membership function to them and computes the uncertainty band of dynamics of TIS agents (cells/cytokines). The list box contains the following contents (Table 1 of paper):

- none (no fuzzy parameters and all the parameters are crisp)
- *a1* (Cancer growth rate)
- *Cmax* (Maximum sustainable tumor cell population)
- *b1* (NK-mediated tumor cell kill rate)
- *c1* (CTL-mediated tumor cell kill rate)
- *d1* (Proportional parameter of anti-CD25 treatment for inhibition of anti-immune effects of Treg on CTL-mediated tumor cell killing)
- *e1* (Rate of suppressive effect of Treg on CTL-mediated tumor cell killing)
- *f1* (Rate of suppressive effect of TGF-β on CTL-mediated tumor cell killing)
- *h1* (Proportional parameter of tumor inhibition rate by Treg depletion through anti-CD25 treatment)
- *k1* (Proportional parameter of tumor inhibition rate by Treg depletion through anti-CD25 treatment)
- *l1* (Depth of access of immune cells to tumor mass)
- *a2* (Constant generation source of NK cells)
- *b2* (Exponential death rate of NK cells)
- *c2* (Maximum of IL-2-mediated NK cell growth)
- *d2* (Steepness coefficient of the IL-2-mediated NK cell growth rate)
- *e2* (Maximum of IFN-γ mediated NK cell growth rate)
- *f2* (The steepness coefficient of the IFN-γ-mediated NK cell growth rate)
- *g2* (Inactivation rate of NK cells by tumor cells)
- *h2* (Suppression rate of NK cells by Tregs)
- *a3* (Exponential death rate of CTLs)
- *b3* (Maximum tumor-mediated CTL recruitment rate)
- *c3* (Steepness coefficient of the tumor-mediated CTL recruitment curve)
- *d3* (CTL stimulation rate by tumor-NK cells interactions)
- *e3* (Inactivation rate of CTLs by tumor cells)
- *f3* (Maximum of IL-2-mediated CTL growth rate)
- *g3* (The steepness coefficient of the IL-2-mediated CTL growth rate)
- *h3* (Maximum of IFN-γ-mediated CTL growth rate)
- *k3* (The steepness coefficient of the IFN-γ-mediated CTL growth rate)
- *l3* (Suppression rate of CTLs by Tregs)
- *n3* (Parameter for MDSC-induced inhibition of CTL proliferation)
- *p3* (Normal number of splenic MDSCs in C57/BL6 mice)
- *m3* (Minimal CTL proliferation factor induced by inhibition of MDSCs)
- *a4* (Normal MDSC production rate)
- *c4* (MDSC expansion coefficient in Panc02 tumor-bearing mice)
- *d4* (Steepness coefficient of the tumor-mediated MDSC production curve)
- *a5* (Production rate of T helper cells in the thymus)
- *b5* (Exponential death rate of T helper cells based on half-life)
- *c5* (Maximum IL-2-mediated T helper cell proliferation rate)
- *d5* (Steepness coefficient of the IL-2-mediated T helper cell proliferation curve)
- *e5* (Maximum IFN-γ-mediated T helper cell proliferation rate)
- *f5* (Steepness coefficient of the IFN-γ-mediated T helper cell proliferation curve)
- *g5* (Suppression rate of T helper cells by Tregs)
- *a6* (Constant production rate of Tregs)
- *b6* (Exponential death rate of Tregs based on half-life)
- *c6* (Treg origination rate from CTLs)
- *d6* (Treg origination rate from T helper cells)
- *e6* (Maximum IL-2-mediated growth rate of Tregs)
- *f6* (Steepness coefficient of the IL-2-mediated Treg growth curve)
- *g6* (NK-mediated Treg degradation constant rate)
- *h6* (Constant inhibition rate of Tregs by anti-CD25 treatment)
- *tau1toalpha1* (Natural death rate of IL-2 based on its half-life/ constant production rate of IL-2 by T helper cells)
- *beta1totau2* (constant production rate of IFN-γ by CTLs/ Natural death rate of IFN-γ based on its half-life)
- *beta2totau2* (Secretion rate of IFN-γ by NK cells/degradation rate of IFN-γ based on its half-life)
- *beta3totau2* (Secretion rate of IFN-γ by T helper cells/degradation rate of IFN-γ based on its half-life)
- *landa1totau3* (Constant production rate of TGF-β by tumor cells/death rate of TGF-β based on its half-life)

After selecting the fuzzy/crisp setting of the model, we can determine the timing of the simulation. We can set the end time of simulation, times of 5-FU, and anti-Treg treatments. For example, in Figure 3, we set time end of simulation: day 150, and times of 5-FU therapy: days 1, 2, 3, and 4 after tumor inoculation and times of anti-Treg therapy: days 3, 6, 10, 13, 17 and 20 after tumor injection.


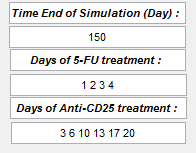


Figure 3. Simulation configuration window. The window consists of the end time end of model simulation, times of 5-FU, and anti-CD25 therapies.

After selection of crisp or fuzzy setting and adjusting timing of model simulation, we clear the workspace of MATLAB by selecting the 'Clear' button, then press the 'RUN' button to simulate TIS with the specified setting.


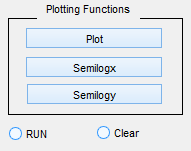


Figure 4. Simulation configuration window. The window consists of the different modalities of the plot including simple plot and logarithmic plot (Y-axis by 'simlogy' and X-axis by 'simlogx’), also two push buttons for clearing (Clear) the workspace of MATLAB and execution of model (RUN).

After the execution of the TIS model, you can see the results of the model simulation by selecting a button: 'update list box'. The simulation outcomes in crisp and fuzzy settings are depicted in Figure 5. A and Figure 5. B, respectively.

| A  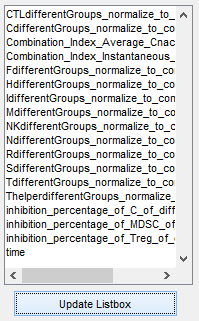 | B  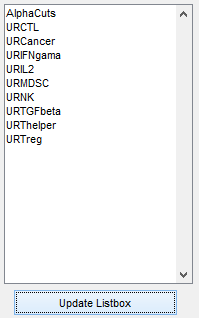 |
| --- | --- |

Figure 5. The list box of outcome measures of the model in the crisp setting of parameters (Figure A) and fuzzy setting of kinetic parameters (Figure B).

The list box of figure 5. A consists of the following outcome measures of the model in the crisp setting of kinetic parameters. In the following, we describe outcome measures of the TIS model are achieved by simulating the model in the specified time setting.

- *CdifferentGroups_normalize_to_control* (the ratio of the population of cancer cells in treatment groups (5-FU, anti-Treg and combination therapy) with respect to the control group during the time)
- *Combination_Index_Average_Cnacer (*the combination index of 5-FU and anti-Treg therapies according to average tumor cell population during the time)
- *Combination_Index_Instantaneous_Cancer* (the combination index of 5-FU and anti-Treg therapies according to instantaneous tumor cell population during the time)
- *FdifferentGroups_normalize_to_control* (the ratio of the concentration of IFN-γ in treatment groups (5-FU, anti-Treg and combination therapy) with respect to the control group during the time)
- *HdifferentGroups_normalize_to_control* (the ratio of the population of T helper (TCD4+) cells in treatment groups (5-FU, anti-Treg and combination therapy) with respect to the control group during the time)
- *IdifferentGroups_normalize_to_control* (the ratio of the concentration of IL-2 in treatment groups (5-FU, anti-Treg and combination therapy) with respect to the control group during the time)
- *MdifferentGroups_normalize_to_control* (the ratio of the population of MDSCs in treatment groups (5-FU, anti-Treg and combination therapy) with respect to the control group during the time)
- *NdifferentGroups_normalize_to_control* (the ratio of the population of NK cells in treatment groups (5-FU, anti-Treg and combination therapy) with respect to the control group during the time)
- *RdifferentGroups_normalize_to_control* (the ratio of the population of Treg cells in treatment groups (5-FU, anti-Treg and combination therapy) with respect to the control group during the time)
- *SdifferentGroups_normalize_to_control* (the ratio of the concentration of TGF-β in treatment groups (5-FU, anti-Treg and combination therapy) with respect to the control group during the time)
- *TdifferentGroups_normalize_to_control* (the ratio of the population of CTLs in treatment groups (5-FU, anti-Treg and combination therapy) with respect to the control group during the time)
- *inhibition_percentage_of_C_of_differentGroups* (inhibition percentage of cancer cells in 5-FU, anti-Treg, and combination therapies during the time)
- *inhibition_percentage_of_MDSC_of_differentGroups* (inhibition percentage of MDSCs in 5-FU, anti-Treg, and combination therapies during the time)
- *inhibition_percentage_of_Treg_of_differentGroups* (inhibition percentage of Tregs in 5-FU, anti-Treg, and combination therapies during the time)
- *time* (time of simulation)

The list box of figure 5.B consist of the following outcome measures of the model in the fuzzy setting of kinetic parameters. In the following, we describe each of the outcome measures of the TIS model is achieved by simulating the model in the specified time setting. The membership function of dynamics of cells/cytokines is computed by averaging dynamics of cells/cytokines from the first injection of drug/drugs until day 100 in treatments groups (including 5-FU, anti-Treg, and combination therapy) and from day 0 to day 100 in no treatment group (control).

- *AlphaCuts* (α-cuts of membership function of fuzzy outcome measures)
- *URCTL* (the membership function of the averaged population of CTLs in the fuzzy setting of kinetic parameters)
- *URCancer* (the membership function of the averaged population of cancer cells in the fuzzy setting of kinetic parameters)
- *URIFNgama* (the membership function of the averaged concentration of IFN-γ in the fuzzy setting of kinetic parameters)
- *URIL2* (the membership function of the averaged concentration of IL-2 in the fuzzy setting of kinetic parameters)
- *URMDSC* (the membership function of the averaged population of MDSCs in the fuzzy setting of kinetic parameters)
- *URNK* (the membership function of the averaged population of NK cells in the fuzzy setting of kinetic parameters)
- *URTGFbeta* (the membership function of the averaged concentration of TGF-β in the fuzzy setting of kinetic parameters)
- *URThelper* (the membership function of the averaged population of T helper cells in the fuzzy setting of kinetic parameters)
- *URTreg* (the membership function of the averaged population of Tregs in the fuzzy setting of kinetic parameters)

In this section we want to simulate TIS, at first, we clear the workspace by the 'Clear' button. Then, by selecting 'none' in the list box of fuzzy parameters and assigning the value 150 to ‘time end of simulation’ and assigning values 1, 2, 3, and 4 for ‘days of 5-FU therapy’ and selecting the values 3, 6, 10, 13, 17 and 20 for ‘days of anti-Treg therapy’, we simulate the model by pushing the pushbutton ‘RUN’. After that by pushing the keyboard 'update', we update the list box of outcome measures of the model. By selecting both ‘time’ and ‘*CdifferentGroups_normalize_to_control* 'as outcome measures and pushing the keyboard 'plot' we can capture dynamics of the relative population of cancer cells in different groups with respect to the control group. The following screenshot depicts the GUI and result of the simulation.


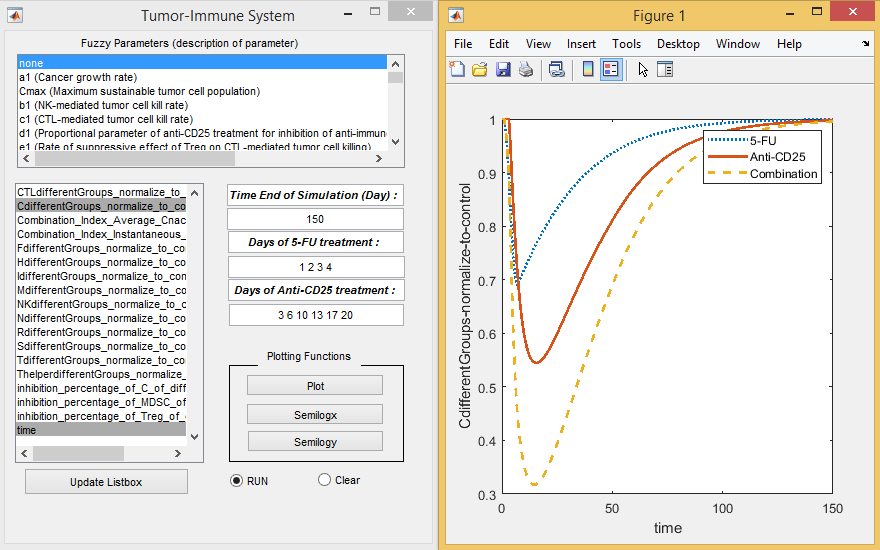


Figure 6. Screenshot for simulating the ODE model of TIS. All kinetic parameters of the model are crisp and the end time of simulation is 150, days of 5-FU therapy are 1, 2, 3, and 4 days after tumor inoculation and days of anti-CD25 therapy are 3, 6, 10, 13, 17, and 20 days after tumor injection. The right figure depicts the ratio of the cancer cell population in treatment groups with respect to the control group.

After simulation of the model, we can use the pushbutton 'clear' to clean the workspace (memory) and by choosing the button 'update list box' we can start the simulation with the new setting. For example, we can select some parameters of list box fuzzy parameters to assign a triangular membership function to them and capture the uncertainty bands of cells or cytokines of TIS.


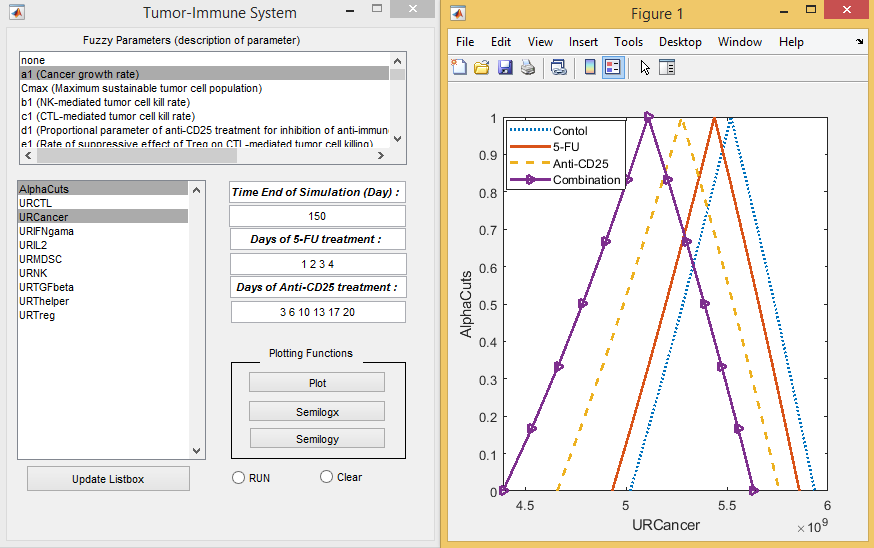


Figure 7. Simulation result for the FODE for control group, 5-FU, anti-Treg, and combination therapies groups. The triangular fuzzy membership function (MF) of cancer cells (average of cancer cells from the start of therapy until time end of simulation) is computed as a result of the uncertainty of kinetic parameter $a_{1}$, in each of control, 5-FU, anti-Treg, and combination therapy groups.

The horizontal axis of Figure 7 (right) depicts the maximum uncertainty band of cancer cells in different groups. As depicted in Figure 7 (right), different treatments caused the uncertainty band of cancer cells to shift to the left (lower population of cancer cells).


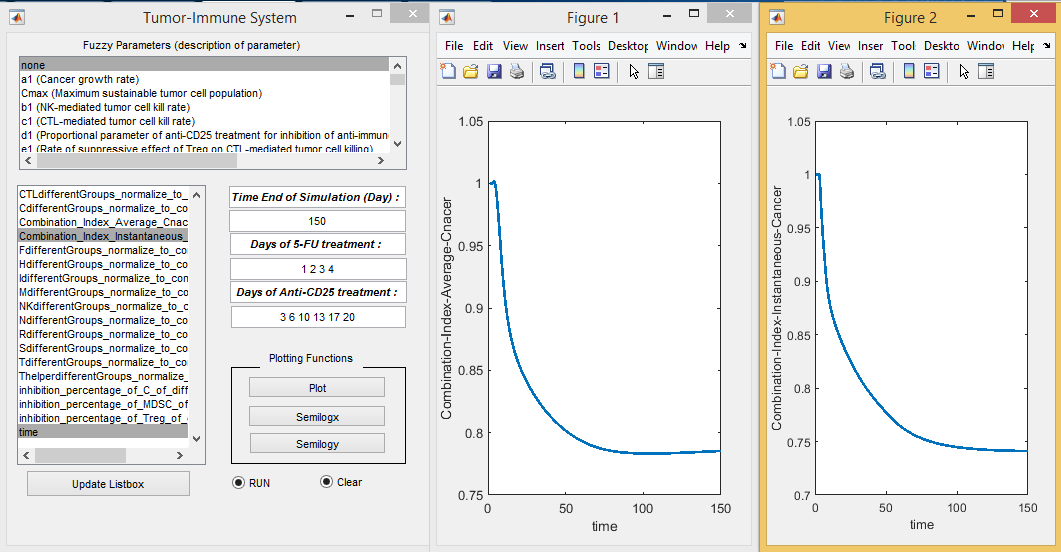


Figure 8. Analysis of interaction among treatments. The dynamics of combination index of 5-FU and anti-CD25 therapies with regarding instantaneous and average tumor size.

**In the following, we provided the MATLAB codes. These codes are attached as supplementary file that are readable by MATLAB software.**

**Function 1: Dynamic_ODE:**

function [C, N, T, M, H, R, I, F, S] = Dynamic_ODE(initial_cond,Time_end, dt,treatment_param,...

is_empty_cd25,is_empty_5fu)

parammmm;

nn = Time_end/dt;

if (~is_empty_5fu) && (~is_empty_cd25)

g_c = treatment_param(:,1);

b_m = treatment_param(:,2);

CTX = treatment_param(:,3);

N_CIK = zeros(nn,1);

elseif (~is_empty_5fu) && (is_empty_cd25)

g_c = treatment_param(:,1);

b_m = treatment_param(:,2);

CTX = zeros(nn,1);

N_CIK = zeros(nn,1);

elseif (is_empty_5fu) && (~is_empty_cd25)

CTX = treatment_param(:,3);

g_c = zeros(nn,1);

b_m = .13*.25*ones(nn,1);

N_CIK = zeros(nn,1);

elseif (is_empty_5fu) && (is_empty_cd25)

CTX = zeros(nn,1);

g_c = zeros(nn,1);

b_m = .13*.25*ones(nn,1);

N_CIK = zeros(nn,1);

end

[ind_CIKtrt,~]=find(N_CIK~=0);

Cstar = zeros(1,nn); CstarwC = zeros(1,nn);

S = zeros(1,nn); rate1_1p = zeros(1,nn);

rate1_2n = zeros(1,nn); rate1_3n = zeros(1,nn);

rate1_4n = zeros(1,nn); rate1_5n = zeros(1,nn);

C = zeros(1,nn);

rate2_1p = zeros(1,nn); rate2_2n = zeros(1,nn);

rate2_3p = zeros(1,nn);rate2_4p = zeros(1,nn);

rate2_5n = zeros(1,nn); rate2_6n = zeros(1,nn);

N = zeros(1,nn);

rate3_1n = zeros(1,nn);rate3_2p = zeros(1,nn);

rate3_3p = zeros(1,nn);rate3_4n = zeros(1,nn);

rate3_5p = zeros(1,nn);rate3_6p = zeros(1,nn);

rate3_7n = zeros(1,nn);

T = zeros(1,nn);

rate4_1p = zeros(1,nn); rate4_2n = zeros(1,nn);

rate4_3p = zeros(1,nn);

M = zeros(1,nn);

rate5_1p = zeros(1,nn); rate5_2n = zeros(1,nn);

rate5_3p = zeros(1,nn); rate5_4p = zeros(1,nn);

rate5_5n = zeros(1,nn);

H = zeros(1,nn);

rate6_1p = zeros(1,nn); rate6_2n = zeros(1,nn);

rate6_3p = zeros(1,nn); rate6_4p = zeros(1,nn);

rate6_5p = zeros(1,nn); rate6_6n = zeros(1,nn);

rate6_7n = zeros(1,nn);

R = zeros(1,nn);

C(1) = initial_cond(1); N(1) = initial_cond(2); T(1) = initial_cond(3); M(1) = initial_cond(4);

H(1) = initial_cond(5); R(1) = initial_cond(6);

for n=1:Time_end/dt-1

if sum(n==ind_CIKtrt)>0

N(n) = max(N_CIK);

end

Cstar(n) = C(n)/(1+(1/l_c)*(abs(C(n)))^(1/3));

CstarwC(n) = 1/(1+(1/l_c)*(abs(C(n)))^(1/3));

S(n) = ((1-m_t)/(1+n_t*(M(n)-p_t)^2))+m_t;

%rates of 1 equ C

rate1_1p(n) = a_c*C(n)*log(Cmax/C(n));

rate1_2n(n) = b_c*N(n)*Cstar(n);

rate1_3n(n) = c_c*T(n)*(1+d_c*CTX(n)^2*(abs(R(n)))^3)*Cstar(n)/( (1+e_c*R(n))*(1+f_c*landa2totau3*C(n)) );

rate1_4n(n) = g_c(n)*C(n);

rate1_5n(n) = h_c*abs(log10(1+ double(CTX(n)>0) *(k_c*CTX(n)*C(n)^1.4)));

C(n+1) = C(n)+dt*(rate1_1p(n)-rate1_2n(n)-rate1_3n(n)-rate1_4n(n)-rate1_5n(n));

if C(n+1)<0

C(n+1) = 1;

end

% rates of 2 equ N

rate2_1p(n) = a_n;

rate2_2n(n) = b_n*N(n);

rate2_3p(n) = c_n*H(n)*N(n)/(d_n*tau1toalpha1+H(n));

rate2_4p(n) = e_n*(beta1totau2*T(n)+beta2totau2*N(n)+beta3totau2*H(n))...

*N(n)/(f_n+(beta1totau2*T(n)+beta2totau2*N(n)+beta3totau2*H(n)));

rate2_5n(n) = g_n*N(n)*Cstar(n);

rate2_6n(n) = h_n*R(n)*N(n);

N(n+1) = N(n)+dt*(rate2_1p(n)-rate2_2n(n)+rate2_3p(n)+rate2_4p(n)-rate2_5n(n)-rate2_6n(n));

if N(n+1)<0

N(n+1) = 1;

end

%rates of 3 equ T

rate3_1n(n) = a_t*T(n);

rate3_2p(n) = b_t*T(n)*C(n)^2/(c_t+C(n)^2);

rate3_3p(n) = d_t*N(n)*Cstar(n)*S(n);

rate3_4n(n) = e_t*T(n)*Cstar(n);

rate3_5p(n) = f_t*H(n)*T(n)/(g_t*...

tau1toalpha1+H(n));

rate3_6p(n) = h_t*(beta1totau2*T(n)+beta2totau2*N(n)+beta3totau2*H(n))...

*T(n)/(k_t+beta1totau2*T(n)+beta2totau2*N(n)+beta3totau2*H(n));

rate3_7n(n) = l_t*R(n)*T(n);

T(n+1) = T(n)+dt*(-rate3_1n(n)+rate3_2p(n)+rate3_3p(n)-rate3_4n(n)+rate3_5p(n)...

+rate3_6p(n)-rate3_7n(n));

if T(n+1)<0

T(n+1) = 1;

end

%rates of 4 equ M

rate4_1p(n) = a_m;

rate4_2n(n) = b_m(n)*M(n);

rate4_3p(n) = c_m*C(n)/(d_m+C(n));

M(n+1) = M(n)+dt*(rate4_1p(n)-rate4_2n(n)+rate4_3p(n));

if M(n+1)<0

M(n+1) = 1;

end

% rates of 6 equ H (TCD4+)

rate5_1p(n) = a_h;

rate5_2n(n) = b_h*H(n);

rate5_3p(n) = c_h*H(n)^2/(d_h*tau1toalpha1+H(n));

rate5_4p(n) = e_h*(beta1totau2*T(n)+beta2totau2*N(n)+...

beta3totau2*H(n))*H(n)/(f_h+(beta1totau2*T(n)+...

beta2totau2*N(n)+beta3totau2*H(n)));

rate5_5n(n) = g_h*R(n)*H(n);

H(n+1) = H(n)+dt*(rate5_1p(n)-rate5_2n(n)+rate5_3p(n)+...

rate5_4p(n)- rate5_5n(n));

if H(n+1)<0

H(n+1) = 1;

end

% rates of 6 equ Treg

rate6_1p(n) = a_tr;

rate6_2n(n) = b_tr*R(n);

rate6_3p(n) = c_tr*T(n);

rate6_4p(n) = d_tr*H(n);

rate6_5p(n) = e_tr*H(n)*R(n)/(f_tr*tau1toalpha1+H(n));

rate6_6n(n) = g_tr*N(n)*R(n);

rate6_7n(n) = h_tr*CTX(n)*R(n);

R(n+1) = R(n)+dt*(rate6_1p(n)-rate6_2n(n)+rate6_3p(n)+...

rate6_4p(n)+rate6_5p(n)-rate6_6n(n)-rate6_7n(n));

if R(n+1)<0

R(n+1) = 1;

end

end

I = (1/tau1toalpha1).*H;

F = beta1totau2.*T+beta2totau2.*N+beta3totau2.*H;

S = landa2totau3.*C;

end

**Function 2: Dynamic_ODE_fuzzy:**

function [C, N, T, M, H, R, I, F, S] = Dynamic_ODE_fuzzy(initial_condition,Time_end,...

dt,treatment_param, ccf,idx,is_empty_cd25,is_empty_5fu)

parammmm;

nn = Time_end/dt;

if (~is_empty_5fu) && (~is_empty_cd25)

g_c = treatment_param(:,1);

b_m = treatment_param(:,2);

CTX = treatment_param(:,3);

N_CIK = zeros(nn,1);

elseif (~is_empty_5fu) && (is_empty_cd25)

g_c = treatment_param(:,1);

b_m = treatment_param(:,2);

CTX = zeros(nn,1);

N_CIK = zeros(nn,1);

elseif (is_empty_5fu) && (~is_empty_cd25)

CTX = treatment_param(:,3);

g_c = zeros(nn,1);

b_m = .13*.25*ones(nn,1);

N_CIK = zeros(nn,1);

elseif (is_empty_5fu) && (is_empty_cd25)

CTX = zeros(nn,1);

g_c = zeros(nn,1);

b_m = .13*.25*ones(nn,1);

N_CIK = zeros(nn,1);

end

param = [a_c Cmax b_c c_c d_c e_c f_c h_c k_c l_c,...

a_n b_n c_n d_n e_n f_n g_n h_n,...

a_t b_t c_t d_t e_t f_t g_t h_t k_t l_t,n_t, p_t, m_t,...

a_m c_m d_m,...

a_h b_h c_h d_h e_h f_h g_h,...

a_tr b_tr c_tr d_tr e_tr f_tr g_tr h_tr,...

tau1toalpha1 beta1totau2 beta2totau2 beta3totau2 landa2totau3];

for itr_fuz=1:numel(idx)

param(idx(itr_fuz)) = ccf(itr_fuz);

end

a_c= param(1); Cmax= param(2); b_c= param(3); c_c= param(4); d_c= param(5); e_c= param(6);

f_c= param(7); h_c= param(8); k_c = param(9); l_c= param(10);

a_n= param(11); b_n= param(12); c_n= param(13); d_n= param(14); e_n= param(15); f_n= param(16);

g_n= param(17); h_n= param(18); a_t= param(19); b_t= param(20); c_t= param(21); d_t= param(22);

e_t= param(23); f_t= param(24); g_t= param(25); h_t= param(26); k_t= param(27); l_t= param(28);

n_t= param(29); p_t= param(30); m_t= param(31); a_m= param(32); c_m= param(33); d_m= param(34);

a_h= param(35); b_h= param(36); c_h= param(37); d_h= param(38); e_h= param(39); f_h= param(40);

g_h= param(41); a_tr= param(42); b_tr= param(43); c_tr= param(44); d_tr= param(45); e_tr= param(46);

f_tr= param(47); g_tr= param(48); h_tr= param(49);tau1toalpha1= param(50); beta1totau2= param(51);

beta2totau2= param(52); beta3totau2= param(53); landa2totau3= param(54);

[ind_CIKtrt,~]=find(N_CIK~=0);

Cstar = zeros(1,nn); CstarwC = zeros(1,nn);

S = zeros(1,nn); rate1_1p = zeros(1,nn);

rate1_2n = zeros(1,nn); rate1_3n = zeros(1,nn);

rate1_4n = zeros(1,nn); rate1_5n = zeros(1,nn);

% rate1_6n = zeros(1,nn);

C = zeros(1,nn);

rate2_1p = zeros(1,nn); rate2_2n = zeros(1,nn);

rate2_3p = zeros(1,nn);rate2_4p = zeros(1,nn);

rate2_5n = zeros(1,nn); rate2_6n = zeros(1,nn);

N = zeros(1,nn);

rate3_1n = zeros(1,nn);rate3_2p = zeros(1,nn);

rate3_3p = zeros(1,nn);rate3_4n = zeros(1,nn);

rate3_5p = zeros(1,nn);rate3_6p = zeros(1,nn);

rate3_7n = zeros(1,nn);

T = zeros(1,nn);

rate4_1p = zeros(1,nn); rate4_2n = zeros(1,nn);

rate4_3p = zeros(1,nn);

M = zeros(1,nn);

rate5_1p = zeros(1,nn); rate5_2n = zeros(1,nn);

rate5_3p = zeros(1,nn); rate5_4p = zeros(1,nn);

rate5_5n = zeros(1,nn);

H = zeros(1,nn);

rate6_1p = zeros(1,nn); rate6_2n = zeros(1,nn);

rate6_3p = zeros(1,nn); rate6_4p = zeros(1,nn);

rate6_5p = zeros(1,nn); rate6_6n = zeros(1,nn);

rate6_7n = zeros(1,nn);

R = zeros(1,nn);

C(1) = initial_condition(1); N(1) = initial_condition(2); T(1) = initial_condition(3);

M(1) = initial_condition(4); H(1) = initial_condition(5); R(1) = initial_condition(6);

for n=1:Time_end/dt-1

if sum(n==ind_CIKtrt)>0

N(n) = max(N_CIK);

end

Cstar(n) = C(n)/(1+(1/l_c)*(abs(C(n)))^(1/3));

CstarwC(n) = 1/(1+(1/l_c)*(abs(C(n)))^(1/3));

S(n) = ((1-m_t)/(1+n_t*(M(n)-p_t)^2))+m_t;

%rates of 1 equ C

rate1_1p(n) = a_c*C(n)*log(Cmax/C(n));

rate1_2n(n) = b_c*N(n)*Cstar(n);

rate1_3n(n) = c_c*T(n)*(1+d_c*CTX(n)^2*(abs(R(n)))^3)*Cstar(n)/( (1+e_c*R(n))*(1+f_c*landa2totau3*C(n)) );

rate1_4n(n) = g_c(n)*C(n);

% rate1_5n(n) = h_c*abs(log10(1+ double(CTX(n)>0) *sum(k_c*CTX(1:n).*C(1:n)')));

rate1_5n(n) = h_c*abs(log10(1+ double(CTX(n)>0) *(k_c*CTX(n)*C(n)^1)));

C(n+1) = C(n)+dt*(rate1_1p(n)-rate1_2n(n)-rate1_3n(n)-rate1_4n(n)-rate1_5n(n));

if C(n+1)<0

C(n+1) = 1;

end

% rates of 2 equ N

rate2_1p(n) = a_n;

rate2_2n(n) = b_n*N(n);

% rate2_3p(n) = g*C(n)^2*N(n)/(h+C(n)^2);

rate2_3p(n) = c_n*H(n)*N(n)/(d_n*tau1toalpha1+H(n));

rate2_4p(n) = e_n*(beta1totau2*T(n)+beta2totau2*N(n)+beta3totau2*H(n))...

*N(n)/(f_n+(beta1totau2*T(n)+beta2totau2*N(n)+beta3totau2*H(n)));

rate2_5n(n) = g_n*N(n)*Cstar(n);

rate2_6n(n) = h_n*R(n)*N(n);

N(n+1) = N(n)+dt*(rate2_1p(n)-rate2_2n(n)+rate2_3p(n)+rate2_4p(n)-rate2_5n(n)-rate2_6n(n));

if N(n+1)<0

N(n+1) = 1;

end

%rates of 3 equ T

rate3_1n(n) = a_t*T(n);

rate3_2p(n) = b_t*T(n)*C(n)^2/(c_t+C(n)^2);

rate3_3p(n) = d_t*N(n)*Cstar(n)*S(n);

rate3_4n(n) = e_t*T(n)*Cstar(n);

rate3_5p(n) = f_t*H(n)*T(n)/(g_t*...

tau1toalpha1+H(n));

rate3_6p(n) = h_t*(beta1totau2*T(n)+beta2totau2*N(n)+beta3totau2*H(n))...

*T(n)/(k_t+beta1totau2*T(n)+beta2totau2*N(n)+beta3totau2*H(n));

rate3_7n(n) = l_t*R(n)*T(n);

T(n+1) = T(n)+dt*(-rate3_1n(n)+rate3_2p(n)+rate3_3p(n)-rate3_4n(n)+rate3_5p(n)...

+rate3_6p(n)-rate3_7n(n));

if T(n+1)<0

T(n+1) = 1;

end

%rates of 4 equ M

rate4_1p(n) = a_m;

rate4_2n(n) = b_m(n)*M(n);

rate4_3p(n) = c_m*C(n)/(d_m+C(n));

M(n+1) = M(n)+dt*(rate4_1p(n)-rate4_2n(n)+rate4_3p(n));

if M(n+1)<0

M(n+1) = 1;

end

% rates of 6 equ H (TCD4+)

rate5_1p(n) = a_h;

rate5_2n(n) = b_h*H(n);

rate5_3p(n) = c_h*H(n)^2/(d_h*tau1toalpha1+H(n));

rate5_4p(n) = e_h*(beta1totau2*T(n)+beta2totau2*N(n)+...

beta3totau2*H(n))*H(n)/(f_h+(beta1totau2*T(n)+...

beta2totau2*N(n)+beta3totau2*H(n)));

rate5_5n(n) = g_h*R(n)*H(n);

H(n+1) = H(n)+dt*(rate5_1p(n)-rate5_2n(n)+rate5_3p(n)+...

rate5_4p(n)- rate5_5n(n));

if H(n+1)<0

H(n+1) = 1;

end

% rates of 6 equ Treg

rate6_1p(n) = a_tr;

rate6_2n(n) = b_tr*R(n);

rate6_3p(n) = c_tr*T(n);

rate6_4p(n) = d_tr*H(n);

rate6_5p(n) = e_tr*H(n)*R(n)/(f_tr*tau1toalpha1+H(n));

rate6_6n(n) = g_tr*N(n)*R(n);

rate6_7n(n) = h_tr*CTX(n)*R(n);

R(n+1) = R(n)+dt*(rate6_1p(n)-rate6_2n(n)+rate6_3p(n)+...

rate6_4p(n)+rate6_5p(n)-rate6_6n(n)-rate6_7n(n));

if R(n+1)<0

R(n+1) = 1;

end

end

I = (1/tau1toalpha1).*H;

F = beta1totau2.*T+beta2totau2.*N+beta3totau2.*H;

S = landa2totau3.*C;

end

**Function 3: list_of_fuzzy_params:**

none

a1

Cmax

b1

c1

d1

e1

f1

h1

k1

l1

a2

b2

c2

d2

e2

f2

g2

h2

a3

b3

c3

d3

e3

f3

g3

h3

k3

l3

n3

p3

m3

a4

c4

d4

a5

b5

c5

d5

e5

f5

g5

a6

b6

c6

d6

e6

f6

g6

h6

tau1toalpha1

beta1totau2

beta2totau2

beta3totau2

landa2totau3

**Function 4: mainFODE:**

function [AlphaCuts, URCancer, URNK, URCTL, URMDSC, URThelper, URTreg,...

URIL2, URIFNgama, URTGFbeta, MM2, MM22] = mainFODE(TimeEnd,Time_5fu,Timecd25,idx)

Time_end = TimeEnd;

idx = idx-1;

dt = .01;

set_paramm;

if ( (isempty(Timecd25)) && (~isempty(Time_5fu)) )

TimeRecord = min(Time_5fu);

elseif ( (isempty(Time_5fu)) && (~isempty(Timecd25)) )

TimeRecord = min(Timecd25);

elseif ( (isempty(Time_5fu)) && (isempty(Timecd25)) )

TimeRecord = dt;

elseif ( (~isempty(Time_5fu)) && (~isempty(Timecd25)) )

TimeRecord = min( min(Timecd25), min(Time_5fu));

end

for itr_treatment =1:4

if itr_treatment == 1

flag_5fu = 0;

flag_antiCd25 = 0;

elseif itr_treatment==2

flag_5fu = 1;

flag_antiCd25 = 0;

elseif itr_treatment==3

flag_5fu = 0;

flag_antiCd25 = 1;

elseif itr_treatment==4

flag_5fu = 1;

flag_antiCd25 = 1;

end

if isempty(Timecd25)

flag_antiCd25 = 0;

is_empty_cd25 = 1;

else

is_empty_cd25 = 0;

end

if isempty(Time_5fu)

flag_5fu = 0;

is_empty_5fu = 1;

else

is_empty_5fu = 0;

end

%% 5FU treatment

days_start_5FU = Time_5fu;

days_end_5FU = days_start_5FU+3;

d = zeros(1,Time_end/dt); %d in no treatment is 0 and 5FU teatment is 0.7

dd = zeros(numel(days_start_5FU),Time_end/dt);

beta = 13*.25*zeros(1,Time_end/dt);%beta in no treatment is 0.25 and 5FU teatment is 0.8

betaaa = zeros(numel(days_start_5FU),Time_end/dt);

for i=1:numel(days_start_5FU)

d(1+days_start_5FU(i)/dt:days_end_5FU(i)/dt) = flag_5fu*.035;

dd(i,1+days_start_5FU(i)/dt:days_end_5FU(i)/dt) = flag_5fu*.035;

beta(1+days_start_5FU(i)/dt:days_end_5FU(i)/dt) = flag_5fu*.08;

betaaa(i,1+days_start_5FU(i)/dt:days_end_5FU(i)/dt) = flag_5fu*.08;

end

if numel(Time_5fu)>1

d = sum(dd);

beta = sum(betaaa);

end

beta(beta==0)=.0325;

N_CIK = zeros(1,Time_end/dt);

N_CIK ([5 15 20 25 30]/dt) = 0*1.8e9;

days_start_CTX = Timecd25;

CTX = zeros(1,Time_end/dt);

CTXX = zeros(numel(days_start_CTX),Time_end/dt);

days_end_CTX = days_start_CTX+28;

for i=1:numel(days_start_CTX)

CTX(1+days_start_CTX(i)/dt:days_end_CTX(i)/dt) = flag_antiCd25*.375e8;

CTXX(i,1+days_start_CTX(i)/dt:days_end_CTX(i)/dt) = flag_antiCd25*.375e8;

end

if numel(days_start_CTX)>1

CTXX = sum(CTXX);

end

initial_cond = [2e6, 3.528e5, 7e5, 1e4, 1.8816e6;

2e6, 4.816e5, 8.736e5, .8e4, 2.1168e4;

2e6, 6.104e5, 1.0472e5, .6e4, 2.352e4];

initial_cond(:,6) = (7.5e-2)*initial_cond(:,5); % Treg = 7.5e-2*CD4+

initial_cond(:,1) = 6e5; % tumor inoculation in (pbs 5fu data)

initial_cond(:,2:end) = initial_cond(:,2:end)*(6e5/(2e6)); % tumor inoculation in (pbs 5fu data)

%%

initial_condition = initial_cond(1,:);

if numel(CTXX)>numel(d)

CTXX(numel(d)+1:end)=[];

end

parammmm;

param = [a_c Cmax b_c c_c d_c e_c f_c h_c k_c l_c,...

a_n b_n c_n d_n e_n f_n g_n h_n,...

a_t b_t c_t d_t e_t f_t g_t h_t k_t l_t,n_t, p_t, m_t,...

a_m c_m d_m,...

a_h b_h c_h d_h e_h f_h g_h,...

a_tr b_tr c_tr d_tr e_tr f_tr g_tr h_tr,...

tau1toalpha1 beta1totau2 beta2totau2 beta3totau2 landa2totau3];

treatment_param = [d' beta' CTXX' N_CIK'];

Cfuzzy = zeros(numel(idx),3);

for itr_fuz = 1:numel(idx)

Cfuzzy(itr_fuz,:) = [.9 1 1.1]*param(idx(itr_fuz));

end

%% the kinetic parameters

%% fuzzy setting

N_fuzzy = numel(idx); %defining the number of fuzzy numbers

KK = 3; % the number of the discretization points for each alpha cut

Levels = 3; % the alpha levels we considered

num_outcome_measure = 9;

xx = zeros(num_outcome_measure,2*(Levels-1)+1);

yy = zeros(num_outcome_measure,2*(Levels-1)+1);

count1 = 1;

[row, column] = size(Cfuzzy);

itr_alpha = 0;

for alpha=0:(1/(Levels-1)):(1-1/(Levels-1))

itr_alpha = itr_alpha+1;

count = 1;

CCf = zeros(KK^N_fuzzy, row);

for i=1:1:row

af= Cfuzzy(i,1);

bf= Cfuzzy(i,2);

c1f= Cfuzzy(i,3);

if bf-af<10^(-10) % if the parameter is a crisp value

sf=af;

for j=1:1:KK^N_fuzzy

CCf(j,i) = sf;

end

else

low = af + alpha*(bf-af);

up = c1f - alpha*(c1f-bf);

j= 1;

while j < KK^N_fuzzy

for mf= low:(up-low)/(KK-1):up

for nnf = 1:1:KK^(count-1)

CCf(j,i) = mf;

j=j+1;

end

end

end

count = count + 1;

end

end

MM1 = zeros(num_outcome_measure,KK^N_fuzzy); MMtotal = zeros(size(MM1));

MM1 = zeros(num_outcome_measure,KK^N_fuzzy);

for jj=1:1:KK^N_fuzzy

ccf = CCf(jj,:);

initial_condition = initial_cond(1,:);

[C, N, T, M, H, R, I, F, S] = Dynamic_ODE_fuzzy(initial_condition,Time_end,...

dt,treatment_param, ccf,idx,is_empty_cd25,is_empty_5fu);

y = [C', N', T', M', H', R', I', F', S'];

[row1, column1] = size(y);

if TimeRecord>Time_end

TimeRecord = round(Time_end/2);

end

MM1(:,jj) = mean( y(TimeRecord/dt:end,:) ); %day 150 of data acquisition

MM2(:,1:4,jj,itr_alpha) = [C' N' T' M'];

end

%obtain the min and max values for each alpha level

for rr=1:1:num_outcome_measure

count2= count1;

smv = MM1(rr,:);

smvsort = sort(smv);

[row2, column2] = size(smvsort);

low = smvsort(1,1);

up = smvsort(1,column2) ;

xx(rr,count2) = low;

yy(rr,count2) = alpha;

count2 = count2+1;

xx(rr,count2) = up;

yy(rr,count2) = alpha;

end

count1 = count1+2;

end

%for alpha = 1;

for i=1:1:row

ccf(1,i) = Cfuzzy(i,2);

end

[C, N, T, M, H, R, I, F, S] = Dynamic_ODE_fuzzy(initial_condition,Time_end,...

dt,treatment_param, ccf,idx,is_empty_cd25,is_empty_5fu);

y = [C', N', T', M', H', R', I', F', S'];

MM22 = [C' N' T' M'];

y21 =mean( y(TimeRecord/dt:end,:) );

xx(:,2*(Levels-1)+1) = y21;

yy(:,2*(Levels-1)+1) = 1;

[rowxx columnxx] = size(xx);

XXX = zeros(rowxx,columnxx);

YYY = zeros(rowxx,columnxx);

for r4=1:1:num_outcome_measure %for each species

xxP = xx(r4,:);

yyP = yy(r4,:);

A1 = xxP';

B1 = yyP';

n = size(A1);

[XX,INDEX] = sort(A1);

for i = 1:n

YY(i) = B1(INDEX(i));

end

XXX(r4,:) = XX;

YYY(r4,:) = YY;

end

% save('matlab1')

XXX_All(:,:,itr_treatment) = XXX;

YYY_All(:,:,itr_treatment) = YYY;

end

AlphaCuts=YYY(1,:);

URCancer = XXX_All(1,:,:);

URNK = XXX_All(2,:,:);

URCTL = XXX_All(3,:,:);

URMDSC = XXX_All(4,:,:);

URThelper = XXX_All(5,:,:);

URTreg = XXX_All(6,:,:);

URIL2 = XXX_All(7,:,:);

URIFNgama = XXX_All(8,:,:);

URTGFbeta = XXX_All(9,:,:);

end

**Function 5: mainModel:**

function [time,...

CdifferentGroups_normalize_to_control,...

NdifferentGroups_normalize_to_control,...

TdifferentGroups_normalize_to_control,...

MdifferentGroups_normalize_to_control,...

HdifferentGroups_normalize_to_control,...

RdifferentGroups_normalize_to_control,...

IdifferentGroups_normalize_to_control,...

FdifferentGroups_normalize_to_control,...

SdifferentGroups_normalize_to_control,...

inhibition_percentage_of_C_of_differentGroups,...

NKdifferentGroups_normalize_to_control,...

CTLdifferentGroups_normalize_to_control,...

inhibition_percentage_of_MDSC_of_differentGroups,...

ThelperdifferentGroups_normalize_to_control,...

inhibition_percentage_of_Treg_of_differentGroups,...

Combination_Index_Instantaneous_Cancer,...

Combination_Index_Average_Cnacer] = mainModel(TimeEnd,Time_5fu,Timecd25,idx)

% x_trt = [1 2 3 4 3 6 10 13 17 20];

% x_trt = [time5fu timecd25];

Time_end = TimeEnd;

dt = .01;

set_paramm;

for itr_treatment =1:4

if itr_treatment == 1

flag_5fu = 0;

flag_antiCd25 = 0;

elseif itr_treatment==2

flag_5fu = 1;

flag_antiCd25 = 0;

elseif itr_treatment==3

flag_5fu = 0;

flag_antiCd25 = 1;

elseif itr_treatment==4

flag_5fu = 1;

flag_antiCd25 = 1;

end

if isempty(Timecd25)

flag_antiCd25 = 0;

is_empty_cd25 = 1;

else

is_empty_cd25 = 0;

end

if isempty(Time_5fu)

flag_5fu = 0;

is_empty_5fu = 1;

else

is_empty_5fu = 0;

end

days_start_5FU = Time_5fu;

days_end_5FU = days_start_5FU+3;

d = zeros(1,Time_end/dt); %d in no treatment is 0 and 5FU teatment is 0.7

dd = zeros(numel(days_start_5FU),Time_end/dt);

beta = .0325*ones(1,Time_end/dt);%beta in no treatment is 0.25 and 5FU teatment is 0.8

betaaa = zeros(numel(days_start_5FU),Time_end/dt);

for i=1:numel(days_start_5FU)

d(1+days_start_5FU(i)/dt:days_end_5FU(i)/dt) = flag_5fu*.035;

dd(i,1+days_start_5FU(i)/dt:days_end_5FU(i)/dt) = flag_5fu*.035;

beta(1+days_start_5FU(i)/dt:days_end_5FU(i)/dt) = flag_5fu*.08;

betaaa(i,1+days_start_5FU(i)/dt:days_end_5FU(i)/dt) = flag_5fu*.08;

end

if numel(Time_5fu)>1

d = sum(dd);

beta = sum(betaaa);

end

beta(beta==0)=.0325;

%% CIK : cytokine unduced killer the number of effective NK cell for each CIK immunotherapy administration is 1.8e9

N_CIK = zeros(1,Time_end/dt);

N_CIK ([5 15 20 25 30]/dt) = 0*1.8e9;

days_start_CTX = Timecd25;

CTX = zeros(1,Time_end/dt);

CTXX = zeros(numel(days_start_CTX),Time_end/dt);

days_end_CTX = days_start_CTX+28;

for i=1:numel(days_start_CTX)

CTX(1+days_start_CTX(i)/dt:days_end_CTX(i)/dt) = flag_antiCd25*.375e8;

CTXX(i,1+days_start_CTX(i)/dt:days_end_CTX(i)/dt) = flag_antiCd25*.375e8;

end

if numel(days_start_CTX)>1

CTXX = sum(CTXX);

end

initial_cond = [2e6, 3.528e5, 7e5, 1e4, 1.8816e6;

2e6, 4.816e5, 8.736e5, .8e4, 2.1168e4;

2e6, 6.104e5, 1.0472e5, .6e4, 2.352e4];

initial_cond(:,6) = (7.5e-2)*initial_cond(:,5); % Treg = 7.5e-2*CD4+

%% % these two lines are for data of pbs 5fu paper

initial_cond(:,1) = 6e5; % tumor inoculation in (pbs 5fu data)

initial_cond(:,2:end) = initial_cond(:,2:end)*(6e5/(2e6)); % tumor inoculation in (pbs 5fu data)

%%

initial_condition = initial_cond(1,:);

if numel(CTXX)>numel(d)

CTXX(numel(d)+1:end)=[];

end

treatment_param = [d' beta' CTXX' N_CIK'];

[C, N, T, M, H, R, I, F, S] = Dynamic_ODE(initial_condition,Time_end, dt,treatment_param,...

is_empty_cd25,is_empty_5fu);

time = dt:dt:Time_end;

CdifferentGroups(itr_treatment,:) = C;

NdifferentGroups(itr_treatment,:) = N;

TdifferentGroups(itr_treatment,:) = T;

MdifferentGroups(itr_treatment,:) = M;

HdifferentGroups(itr_treatment,:) = H;

RdifferentGroups(itr_treatment,:) = R;

IdifferentGroups(itr_treatment,:) = I;

FdifferentGroups(itr_treatment,:) = F;

SdifferentGroups(itr_treatment,:) = S;

end

%%

CdifferentGroups_normalize_to_control = CdifferentGroups(2:end,:)./CdifferentGroups(1,:);

NdifferentGroups_normalize_to_control = NdifferentGroups(2:end,:)./NdifferentGroups(1,:);

TdifferentGroups_normalize_to_control = TdifferentGroups(2:end,:)./TdifferentGroups(1,:);

MdifferentGroups_normalize_to_control = MdifferentGroups(2:end,:)./MdifferentGroups(1,:);

HdifferentGroups_normalize_to_control = HdifferentGroups(2:end,:)./HdifferentGroups(1,:);

RdifferentGroups_normalize_to_control = RdifferentGroups(2:end,:)./RdifferentGroups(1,:);

IdifferentGroups_normalize_to_control = IdifferentGroups(2:end,:)./IdifferentGroups(1,:);

FdifferentGroups_normalize_to_control = FdifferentGroups(2:end,:)./FdifferentGroups(1,:);

SdifferentGroups_normalize_to_control = SdifferentGroups(2:end,:)./SdifferentGroups(1,:);

inhibition_percentage_of_C_of_5FU = 100*(1-CdifferentGroups_normalize_to_control(1,:));

inhibition_percentage_of_C_of_AntiCd25 = 100*(1-CdifferentGroups_normalize_to_control(2,:));

inhibition_percentage_of_C_of_combination = 100*(1-CdifferentGroups_normalize_to_control(3,:));

inhibition_percentage_of_C_of_differentGroups = [inhibition_percentage_of_C_of_5FU;

inhibition_percentage_of_C_of_AntiCd25;

inhibition_percentage_of_C_of_combination];

relative_NK_of_5FU_to_NK_of_control = NdifferentGroups_normalize_to_control(1,:);

relative_NK_of_AntiCD25_to_NK_of_control = NdifferentGroups_normalize_to_control(2,:);

relative_NK_of_Combination_to_NK_of_control = NdifferentGroups_normalize_to_control(3,:);

NKdifferentGroups_normalize_to_control = [relative_NK_of_5FU_to_NK_of_control;

relative_NK_of_AntiCD25_to_NK_of_control;

relative_NK_of_Combination_to_NK_of_control];

relative_CTL_of_5FU_to_CTL_of_control = TdifferentGroups_normalize_to_control(1,:);

relative_CTL_of_AntiCD25_to_CTL_of_control = TdifferentGroups_normalize_to_control(2,:);

relative_CTL_of_Combination_to_CTL_of_control = TdifferentGroups_normalize_to_control(3,:);

CTLdifferentGroups_normalize_to_control = [relative_CTL_of_5FU_to_CTL_of_control;

relative_CTL_of_AntiCD25_to_CTL_of_control;

relative_CTL_of_Combination_to_CTL_of_control];

inhibition_percentage_of_MDSC_of_5FU = 100*(1-MdifferentGroups_normalize_to_control(1,:));

inhibition_percentage_of_MDSC_of_AntiCd25 = 100*(1-MdifferentGroups_normalize_to_control(2,:));

inhibition_percentage_of_MDSC_of_combination = 100*(1-MdifferentGroups_normalize_to_control(3,:));

inhibition_percentage_of_MDSC_of_differentGroups = [inhibition_percentage_of_MDSC_of_5FU;

inhibition_percentage_of_MDSC_of_AntiCd25;

inhibition_percentage_of_MDSC_of_combination];

relative_Thelper_of_5FU_to_Thelper_of_control = HdifferentGroups_normalize_to_control(1,:);

relative_Thelper_of_AntiCD25_to_Thelper_of_control = HdifferentGroups_normalize_to_control(2,:);

relative_Thelper_of_Combination_to_Thelper_of_control = HdifferentGroups_normalize_to_control(3,:);

ThelperdifferentGroups_normalize_to_control = [relative_Thelper_of_5FU_to_Thelper_of_control;

relative_Thelper_of_AntiCD25_to_Thelper_of_control;

relative_Thelper_of_Combination_to_Thelper_of_control];

inhibition_percentage_of_Treg_of_5FU = 100*(1-RdifferentGroups_normalize_to_control(1,:));

inhibition_percentage_of_Treg_of_AntiCd25 = 100*(1-RdifferentGroups_normalize_to_control(2,:));

inhibition_percentage_of_Treg_of_combination = 100*(1-RdifferentGroups_normalize_to_control(3,:));

inhibition_percentage_of_Treg_of_differentGroups = [inhibition_percentage_of_Treg_of_5FU;

inhibition_percentage_of_Treg_of_AntiCd25;

inhibition_percentage_of_Treg_of_combination];

Ctreatment_to_control_inst_n = CdifferentGroups(2:end,:)./CdifferentGroups(1,:);

Combination_Index_Instantaneous_Cancer = ( 1-Ctreatment_to_control_inst_n(1,:)+1-Ctreatment_to_control_inst_n(2,:)-...

(1- Ctreatment_to_control_inst_n(1,:) ).*(1- Ctreatment_to_control_inst_n(2,:) ))./...

(1-Ctreatment_to_control_inst_n(3,:));

Ctreatment_to_control_ave1 = cumsum(CdifferentGroups(1,:))./(1:numel(time));

Ctreatment_to_control_ave2 = cumsum(CdifferentGroups(2,:))./(1:numel(time));

Ctreatment_to_control_ave3 = cumsum(CdifferentGroups(3,:))./(1:numel(time));

Ctreatment_to_control_ave4 = cumsum(CdifferentGroups(4,:))./(1:numel(time));

Ctreatment_to_control_ave = [Ctreatment_to_control_ave1;Ctreatment_to_control_ave2;...

Ctreatment_to_control_ave3;Ctreatment_to_control_ave4];

Ctreatment_to_control_ave_n = Ctreatment_to_control_ave(2:end,:)./Ctreatment_to_control_ave(1,:);

Combination_Index_Average_Cnacer = ( 1-Ctreatment_to_control_ave_n(1,:)+1-Ctreatment_to_control_ave_n(2,:)-...

(1-Ctreatment_to_control_ave_n(1,:)).*(1-Ctreatment_to_control_ave_n(2,:) ) )./...

(1-Ctreatment_to_control_ave_n(3,:));

end

**Function 6: parammmm:**

a_c = 0.0440;

Cmax = 1e10;

b_c = 3.23e-7;c_c = 1.1e-7; d_c = 2e-34;

e_c = .345; f_c = .286; h_c = 2.5e5; k_c = 1e-15;

l_c = 100;

a_n = 1.4e4;b_n = 4.12e-2; c_n = 100*.125;

d_n = .3; e_n = 100*.125; f_n = .3; g_n = .01*1e-7;

h_n = 1e-10;

a_t = 2e-2; b_t = .8*1e-1;

c_t = 1e7*2.02e7; d_t = 1.1e-7; e_t = 1.5*1e-10;

f_t = .01*.125; g_t = .3;

h_t = .125; k_t = .3; l_t = 1e-10;

n_t = 6e-3; p_t = 2.5e6; m_t = .18;

a_m = .25*5e6;

c_m = .7e7;

d_m = 1e10;

a_h = 3.6e5; b_h = 1.2e-3;

c_h = .125; d_h = .3;

e_h = .125; f_h = .3;

g_h = 1e-10;

a_tr = 5.6e5; b_tr = .023; c_tr = 2e-4;

d_tr = 4e-4; e_tr = .125; f_tr = .3;

g_tr = 1e-11; h_tr = 1.5e-11;

tau1toalpha1 = 2.2483e11;

beta1totau2 = 4.4691e-13;

beta2totau2 = 4.4691e-13;

beta3totau2 = 4.4691e-13;

landa2totau3 = 2*4.4691e-13;

param = [a_c Cmax b_c c_c d_c e_c f_c h_c k_c l_c,...

a_n b_n c_n d_n e_n f_n g_n h_n,...

a_t b_t c_t d_t e_t f_t g_t h_t k_t l_t,n_t, p_t, m_t,...

a_m c_m d_m,...

a_h b_h c_h d_h e_h f_h g_h,...

a_tr b_tr c_tr d_tr e_tr f_tr g_tr h_tr,...

tau1toalpha1 beta1totau2 beta2totau2 beta3totau2 landa2totau3];

**Function 6: TIS_sa:**

function varargout = TIS_sa(varargin)

% PLOTVARIABLESGUIDEEXAMPLE Application M-file for PlotVariablesGUIDEExample.fig

% PLOTVARIABLESGUIDEEXAMPLE, by itself, creates a new PLOTVARIABLESGUIDEEXAMPLE or raises the existing

% singleton*.

%

% H = PLOTVARIABLESGUIDEEXAMPLE returns the handle to a new PLOTVARIABLESGUIDEEXAMPLE or the handle to

% the existing singleton*.

%

% PLOTVARIABLESGUIDEEXAMPLE('CALLBACK',hObject,eventData,handles,...) calls the local

% function named CALLBACK in PLOTVARIABLESGUIDEEXAMPLE.M with the given input arguments.

%

% PLOTVARIABLESGUIDEEXAMPLE('Property','Value',...) creates a new PLOTVARIABLESGUIDEEXAMPLE or raises the

% existing singleton*. Starting from the left, property value pairs are

% applied to the GUI before lb_OpeningFunction gets called. An

% unrecognized property name or invalid value makes property application

% stop. All inputs are passed to PlotVariablesGUIDEExample_OpeningFcn via varargin.

%

% *See GUI Options - GUI allows only one instance to run (singleton).

%

% See also: GUIDE, GUIDATA, GUIHANDLES

% Edit the above text to modify the response to help PlotVariablesGUIDEExample

% Copyright 2000-2006 The MathWorks, Inc.

% Last Modified by GUIDE v2.5 29-Oct-2019 21:59:50

% Begin initialization code - DO NOT EDIT

gui_Singleton = 1;

gui_State = struct('gui_Name', mfilename, ...

'gui_Singleton', gui_Singleton, ...

'gui_OpeningFcn', @PlotVariablesGUIDEExample_OpeningFcn, ...

'gui_OutputFcn', @PlotVariablesGUIDEExample_OutputFcn, ...

'gui_LayoutFcn', [], ...

'gui_Callback', []);

if nargin && ischar(varargin{1})

gui_State.gui_Callback = str2func(varargin{1});

end

if nargout

[varargout{1:nargout}] = gui_mainfcn(gui_State, varargin{:});

else

gui_mainfcn(gui_State, varargin{:});

end

% End initialization code - DO NOT EDIT

% --- Executes just before PlotVariablesGUIDEExample is made visible.

function PlotVariablesGUIDEExample_OpeningFcn(hObject, eventdata, handles, varargin)

% This function has no output args, see OutputFcn.

% hObject handle to figure

% eventdata reserved - to be defined in a future version of MATLAB

% handles structure with handles and user data (see GUIDATA)

% varargin command line arguments to PlotVariablesGUIDEExample (see VARARGIN)

% Choose default command line output for PlotVariablesGUIDEExample

handles.output = hObject;

% Update handles structure

guidata(hObject, handles);

% Populate the listbox

update_listbox(handles)

set(handles.listbox1,'Value',[])

handles.figure1.Name='Tumor-Immune System';

% UIWAIT makes PlotVariablesGUIDEExample wait for user response (see UIRESUME)

% uiwait(handles.figure1);

% --- Outputs from this function are returned to the command line.

function varargout = PlotVariablesGUIDEExample_OutputFcn(hObject, eventdata, handles)

% varargout cell array for returning output args (see VARARGOUT);

% hObject handle to figure

% eventdata reserved - to be defined in a future version of MATLAB

% handles structure with handles and user data (see GUIDATA)

% Get default command line output from handles structure

varargout{1} = handles.output;

function update_button_Callback(hObject, eventdata, handles)

% hObject handle to update_button (see GCBO)

% eventdata reserved - to be defined in a future version of MATLAB

% handles structure with handles and user data (see GUIDATA)

update_listbox(handles)

function update_listbox(handles)

% hObject handle to update (see GCBO)

% eventdata reserved - to be defined in a future version of MATLAB

% handles structure with handles and user data (see GUIDATA)

% Hints: contents = get(hObject,'String') returns listbox1 contents as cell array

% contents{get(hObject,'Value')} returns selected item from listbox1

% Updates the listbox to match the current workspace

vars = evalin('base','who');

set(handles.listbox1,'String',vars)

function [var1,var2] = get_var_names(handles)

% Returns the names of the two variables to plot

list_entries = get(handles.listbox1,'String');

list_entries4Plot = list_entries;

for itr_list = 1:numel(list_entries)

ind4plot = (find(list_entries4Plot{itr_list}=='_'));

list_entries4Plot{itr_list}(ind4plot)=['-'];

end

index_selected = get(handles.listbox1,'Value');

var1 = [];

var2 = [];

if length(index_selected) ~= 2

errordlg('You must select two variables','Incorrect Selection','modal')

else

var1 = list_entries{index_selected(1)};

var2 = list_entries{index_selected(2)};

end

function plot_button_Callback(hObject, eventdata, handles)

% hObject handle to plot_button (see GCBO)

% eventdata reserved - to be defined in a future version of MATLAB

% handles structure with handles and user data (see GUIDATA)

TimeEnd = str2num(get(handles.Time_end,'string'));

Time_5fu = str2num(get(handles.time5fu,'string'));

Timecd25 = str2num(get(handles.timecd25,'string'));

idx = (get(handles.listbox_fuzzy,'value') );

[x,y] = get_var_names(handles);

xname = x;

xname(find(xname=='_'))=['-'];

yname = y;

yname(find(yname=='_'))=['-'];

if isempty(x) && isempty(y)

return

end

% figure(gcf)

strpp = {':','-','--','->','-o'};

try

if (idx==1)

time = evalin('base',y);

outcome_measure = evalin('base',x);

if size(outcome_measure,1)==1

% evalin('base',['plot(',y,',',x,')']),...

plot(time, outcome_measure,'LineWidth',2),...

xlabel(yname),ylabel(xname), box on;

elseif size(outcome_measure,1)==3

for itr_pp=1:size(outcome_measure,1)

plot(time, outcome_measure(itr_pp,:),strpp{itr_pp},'LineWidth',2), hold on;

legend('5-FU','Anti-CD25','Combination'), xlabel(yname),ylabel(xname), box on;

end

elseif size(outcome_measure,1)==4

for itr_pp=1:size(outcome_measure,3)

plot(time, outcome_measure(itr_pp,:),strpp{itr_pp},'LineWidth',2), hold on;

legend('Contol','5-FU','Anti-CD25','Combination'), xlabel(yname),ylabel(xname), box on;

end

end

else

time = evalin('base',x);

outcome_measure = evalin('base',y);

for itr_pp=4:size(outcome_measure,3)

plot(outcome_measure(:,:,itr_pp),time,strpp{1},'LineWidth',2),hold on;

legend('Contol','5-FU','Anti-CD25','Combination'), ...

ylabel('AlphaCuts'),xlabel(yname), box on;

end

% evalin('base',['plot(',y,',',x,')']),...

end

catch ex

errordlg(...

ex.getReport('basic'),'Error generating linear plot','modal')

end

function semilogx_button_Callback(hObject, eventdata, handles)

% hObject handle to semilogx_button (see GCBO)

% eventdata reserved - to be defined in a future version of MATLAB

% handles structure with handles and user data (see GUIDATA)

idx = (get(handles.listbox_fuzzy,'value') );

[x,y] = get_var_names(handles);

xname = x;

xname(find(xname=='_'))=['-'];

yname = y;

yname(find(yname=='_'))=['-'];

if isempty(x) && isempty(y)

return

end

time = evalin('base',y);

outcome_measure = evalin('base',x);

figure(gcf)

try

if (idx==1)

time = evalin('base',y);

outcome_measure = evalin('base',x);

if size(outcome_measure,1)==1

% evalin('base',['plot(',y,',',x,')']),...

semilogx(time, outcome_measure,'LineWidth',2),...

xlabel(yname),ylabel(xname), box on;

elseif size(outcome_measure,1)==3

for itr_pp=1:size(outcome_measure,1)

semilogx(time, outcome_measure(itr_pp,:),'LineWidth',2),hold on;

legend('5-FU','Anti-CD25','Combination'), xlabel(yname),ylabel(xname), box on;

end

elseif size(outcome_measure,1)==4

for itr_pp=1:size(outcome_measure,1)

semilogx(time, outcome_measure(itr_pp,:),'LineWidth',2),hold on, box on;

legend('Contol','5-FU','Anti-CD25','Combination'), xlabel(yname),ylabel(xname);

end

end

else

time = evalin('base',x);

outcome_measure = evalin('base',y);

for itr_pp=1:size(outcome_measure,3)

semilogx(outcome_measure(:,:,itr_pp),time,'LineWidth',2), hold on;

legend('Contol','5-FU','Anti-CD25','Combination'), ylabel('AlphaCuts'),xlabel(yname), box on;

end

% evalin('base',['plot(',y,',',x,')']),...

end

catch ex

errordlg(...

ex.getReport('basic'),'Error generating semilogx plot','modal')

end

function semilogy_button_Callback(hObject, eventdata, handles)

% hObject handle to semilogy_button (see GCBO)

% eventdata reserved - to be defined in a future version of MATLAB

% handles structure with handles and user data (see GUIDATA)

idx = (get(handles.listbox_fuzzy,'value') );

[x,y] = get_var_names(handles);

xname = x;

xname(find(xname=='_'))=['-'];

yname = y;

yname(find(yname=='_'))=['-'];

if isempty(x) && isempty(y)

return

end

time = evalin('base',y);

outcome_measure = evalin('base',x);

figure(gcf)

try

if (idx==1)

time = evalin('base',y);

outcome_measure = evalin('base',x);

if size(outcome_measure,1)==1

% evalin('base',['plot(',y,',',x,')']),...

semilogy(time, outcome_measure,'LineWidth',2),...

xlabel(yname),ylabel(xname), box on;

elseif size(outcome_measure,1)==3

for itr_pp=1:size(outcome_measure,1)

semilogy(time, outcome_measure(itr_pp,:),'LineWidth',2), hold on, box on;

legend('5-FU','Anti-CD25','Combination'), xlabel(yname),ylabel(xname);

end

elseif size(outcome_measure,1)==4

for itr_pp=1:size(outcome_measure,1)

semilogy(time, outcome_measure(itr_pp,:),'LineWidth',2), hold on, box on;

legend('Contol','5-FU','Anti-CD25','Combination'), xlabel(yname),ylabel(xname);

end

end

else

time = evalin('base',x);

outcome_measure = evalin('base',y);

for itr_pp=1:size(outcome_measure,3)

semilogy(outcome_measure(:,:,itr_pp),time,'LineWidth',2), hold on, box on;

legend('Contol','5-FU','Anti-CD25','Combination'), ylabel('AlphaCuts'),xlabel(yname), box on;

end

% evalin('base',['plot(',y,',',x,')']),...

end

catch ex

errordlg(...

ex.getReport('basic'),'Error generating semilogy plot','modal')

end

% --- Executes during object creation, after setting all properties.

function listbox1_CreateFcn(hObject, eventdata, handles)

% hObject handle to listbox1 (see GCBO)

% eventdata reserved - to be defined in a future version of MATLAB

% handles empty - handles not created until after all CreateFcns called

% Hint: listbox controls usually have a white background, change

% 'usewhitebg' to 0 to use default. See ISPC and COMPUTER.

usewhitebg = ispc;

if usewhitebg

set(hObject,'BackgroundColor','white');

else

set(hObject,'BackgroundColor',get(groot,'defaultUicontrolBackgroundColor'));

end

function Run_Callback(hObject, eventdata, handles)

% hObject handle to Run (see GCBO)

% eventdata reserved - to be defined in a future version of MATLAB

% handles structure with handles and user data (see GUIDATA)

% Hints: get(hObject,'String') returns contents of Run as text

% str2double(get(hObject,'String')) returns contents of Run as a double

% timecd25 = str2num(get(handles.timecd25,'string'));

% time5fu = str2num(get(handles.time5fu,'string'));

% Time_end = str2num(get(handles.Time_end,'string'));

% save('hObject','hObject'); save('handles','handles'); save('eventdata','eventdata');

% evalin( 'base', 'clearvars *' ), close all;

% load('hObject'); load('handles'); load('eventdata');

TimeEnd = str2num(get(handles.Time_end,'string'));

Time_5fu = str2num(get(handles.time5fu,'string'));

Timecd25 = str2num(get(handles.timecd25,'string'));

idx = (get(handles.listbox_fuzzy,'value') );

if (idx==1)

[time,...

CdifferentGroups_normalize_to_control,...

NdifferentGroups_normalize_to_control,...

TdifferentGroups_normalize_to_control,...

MdifferentGroups_normalize_to_control,...

HdifferentGroups_normalize_to_control,...

RdifferentGroups_normalize_to_control,...

IdifferentGroups_normalize_to_control,...

FdifferentGroups_normalize_to_control,...

SdifferentGroups_normalize_to_control,...

inhibition_percentage_of_C_of_differentGroups,...

NKdifferentGroups_normalize_to_control,...

CTLdifferentGroups_normalize_to_control,...

inhibition_percentage_of_MDSC_of_differentGroups,...

ThelperdifferentGroups_normalize_to_control,...

inhibition_percentage_of_Treg_of_differentGroups,...

Combination_Index_Instantaneous_Cancer,...

Combination_Index_Average_Cnacer] = mainModel(TimeEnd,Time_5fu,Timecd25,idx);

assignin('base','time',time);

assignin('base','IdifferentGroups_normalize_to_control',IdifferentGroups_normalize_to_control);

assignin('base','FdifferentGroups_normalize_to_control',FdifferentGroups_normalize_to_control);

assignin('base','SdifferentGroups_normalize_to_control',SdifferentGroups_normalize_to_control);

assignin('base','inhibition_percentage_of_C_of_differentGroups',inhibition_percentage_of_C_of_differentGroups);

assignin('base','NKdifferentGroups_normalize_to_control',NKdifferentGroups_normalize_to_control);

assignin('base','CTLdifferentGroups_normalize_to_control',CTLdifferentGroups_normalize_to_control);

assignin('base','inhibition_percentage_of_MDSC_of_differentGroups',inhibition_percentage_of_MDSC_of_differentGroups);

assignin('base','ThelperdifferentGroups_normalize_to_control',ThelperdifferentGroups_normalize_to_control);

assignin('base','inhibition_percentage_of_Treg_of_differentGroups',inhibition_percentage_of_Treg_of_differentGroups);

assignin('base','CdifferentGroups_normalize_to_control',CdifferentGroups_normalize_to_control);

assignin('base','NdifferentGroups_normalize_to_control',NdifferentGroups_normalize_to_control);

assignin('base','TdifferentGroups_normalize_to_control',TdifferentGroups_normalize_to_control);

assignin('base','MdifferentGroups_normalize_to_control',MdifferentGroups_normalize_to_control);

assignin('base','HdifferentGroups_normalize_to_control',HdifferentGroups_normalize_to_control);

assignin('base','RdifferentGroups_normalize_to_control',RdifferentGroups_normalize_to_control);

assignin('base','Combination_Index_Average_Cnacer',Combination_Index_Average_Cnacer);

assignin('base','Combination_Index_Instantaneous_Cancer',Combination_Index_Instantaneous_Cancer);

else

[AlphaCuts, URCancer, URNK, URCTL, URMDSC, URThelper, URTreg,...

URIL2, URIFNgama, URTGFbeta, MM2, MM22] = mainFODE(TimeEnd,Time_5fu,Timecd25,idx);

assignin('base','AlphaCuts',AlphaCuts);

assignin('base','URCancer',URCancer);

assignin('base','URNK',URNK);

assignin('base','URCTL',URCTL);

assignin('base','URMDSC',URMDSC);

assignin('base','URThelper',URThelper);

assignin('base','URTreg',URTreg);

assignin('base','URIL2',URIL2);

assignin('base','URIFNgama',URIFNgama);

assignin('base','URTGFbeta',URTGFbeta);

end

% --- Executes during object creation, after setting all properties.

function Run_CreateFcn(hObject, eventdata, handles)

% hObject handle to Run (see GCBO)

% eventdata reserved - to be defined in a future version of MATLAB

% handles empty - handles not created until after all CreateFcns called

% Hint: edit controls usually have a white background on Windows.

% See ISPC and COMPUTER.

if ispc && isequal(get(hObject,'BackgroundColor'), get(0,'defaultUicontrolBackgroundColor'))

set(hObject,'BackgroundColor','white');

end

function Time_end_Callback(hObject, eventdata, handles)

% hObject handle to Time_end (see GCBO)

% eventdata reserved - to be defined in a future version of MATLAB

% handles structure with handles and user data (see GUIDATA)

% Hints: get(hObject,'String') returns contents of Time_end as text

% str2double(get(hObject,'String')) returns contents of Time_end as a double

% --- Executes during object creation, after setting all properties.

function Time_end_CreateFcn(hObject, eventdata, handles)

% hObject handle to Time_end (see GCBO)

% eventdata reserved - to be defined in a future version of MATLAB

% handles empty - handles not created until after all CreateFcns called

% Hint: edit controls usually have a white background on Windows.

% See ISPC and COMPUTER.

if ispc && isequal(get(hObject,'BackgroundColor'), get(0,'defaultUicontrolBackgroundColor'))

set(hObject,'BackgroundColor','white');

end

function time5fu_Callback(hObject, eventdata, handles)

% hObject handle to time5fu (see GCBO)

% eventdata reserved - to be defined in a future version of MATLAB

% handles structure with handles and user data (see GUIDATA)

% Hints: get(hObject,'String') returns contents of time5fu as text

% str2double(get(hObject,'String')) returns contents of time5fu as a double

% --- Executes during object creation, after setting all properties.

function time5fu_CreateFcn(hObject, eventdata, handles)

% hObject handle to time5fu (see GCBO)

% eventdata reserved - to be defined in a future version of MATLAB

% handles empty - handles not created until after all CreateFcns called

% Hint: edit controls usually have a white background on Windows.

% See ISPC and COMPUTER.

if ispc && isequal(get(hObject,'BackgroundColor'), get(0,'defaultUicontrolBackgroundColor'))

set(hObject,'BackgroundColor','white');

end

function timecd25_Callback(hObject, eventdata, handles)

% hObject handle to timecd25 (see GCBO)

% eventdata reserved - to be defined in a future version of MATLAB

% handles structure with handles and user data (see GUIDATA)

% Hints: get(hObject,'String') returns contents of timecd25 as text

% str2double(get(hObject,'String')) returns contents of timecd25 as a double

% --- Executes during object creation, after setting all properties.

function timecd25_CreateFcn(hObject, eventdata, handles)

% hObject handle to timecd25 (see GCBO)

% eventdata reserved - to be defined in a future version of MATLAB

% handles empty - handles not created until after all CreateFcns called

% Hint: edit controls usually have a white background on Windows.

% See ISPC and COMPUTER.

if ispc && isequal(get(hObject,'BackgroundColor'), get(0,'defaultUicontrolBackgroundColor'))

set(hObject,'BackgroundColor','white');

end

% --- Executes during object creation, after setting all properties.

function figure1_CreateFcn(hObject, eventdata, handles)

% hObject handle to figure1 (see GCBO)

% eventdata reserved - to be defined in a future version of MATLAB

% handles empty - handles not created until after all CreateFcns called

function edit6_Callback(hObject, eventdata, handles)

% hObject handle to time5fu (see GCBO)

% eventdata reserved - to be defined in a future version of MATLAB

% handles structure with handles and user data (see GUIDATA)

% Hints: get(hObject,'String') returns contents of time5fu as text

% str2double(get(hObject,'String')) returns contents of time5fu as a double

% --- Executes during object creation, after setting all properties.

function edit6_CreateFcn(hObject, eventdata, handles)

% hObject handle to time5fu (see GCBO)

% eventdata reserved - to be defined in a future version of MATLAB

% handles empty - handles not created until after all CreateFcns called

% Hint: edit controls usually have a white background on Windows.

% See ISPC and COMPUTER.

if ispc && isequal(get(hObject,'BackgroundColor'), get(0,'defaultUicontrolBackgroundColor'))

set(hObject,'BackgroundColor','white');

end

function edit8_Callback(hObject, eventdata, handles)

% hObject handle to edit8 (see GCBO)

% eventdata reserved - to be defined in a future version of MATLAB

% handles structure with handles and user data (see GUIDATA)

% Hints: get(hObject,'String') returns contents of edit8 as text

% str2double(get(hObject,'String')) returns contents of edit8 as a double

% --- Executes during object creation, after setting all properties.

function edit8_CreateFcn(hObject, eventdata, handles)

% hObject handle to edit8 (see GCBO)

% eventdata reserved - to be defined in a future version of MATLAB

% handles empty - handles not created until after all CreateFcns called

% Hint: edit controls usually have a white background on Windows.

% See ISPC and COMPUTER.

if ispc && isequal(get(hObject,'BackgroundColor'), get(0,'defaultUicontrolBackgroundColor'))

set(hObject,'BackgroundColor','white');

end

function edit9_Callback(hObject, eventdata, handles)

% hObject handle to edit9 (see GCBO)

% eventdata reserved - to be defined in a future version of MATLAB

% handles structure with handles and user data (see GUIDATA)

% Hints: get(hObject,'String') returns contents of edit9 as text

% str2double(get(hObject,'String')) returns contents of edit9 as a double

% --- Executes during object creation, after setting all properties.

function edit9_CreateFcn(hObject, eventdata, handles)

% hObject handle to edit9 (see GCBO)

% eventdata reserved - to be defined in a future version of MATLAB

% handles empty - handles not created until after all CreateFcns called

% Hint: edit controls usually have a white background on Windows.

% See ISPC and COMPUTER.

if ispc && isequal(get(hObject,'BackgroundColor'), get(0,'defaultUicontrolBackgroundColor'))

set(hObject,'BackgroundColor','white');

end

function edit10_Callback(hObject, eventdata, handles)

% hObject handle to edit10 (see GCBO)

% eventdata reserved - to be defined in a future version of MATLAB

% handles structure with handles and user data (see GUIDATA)

% Hints: get(hObject,'String') returns contents of edit10 as text

% str2double(get(hObject,'String')) returns contents of edit10 as a double

% --- Executes during object creation, after setting all properties.

function edit10_CreateFcn(hObject, eventdata, handles)

% hObject handle to edit10 (see GCBO)

% eventdata reserved - to be defined in a future version of MATLAB

% handles empty - handles not created until after all CreateFcns called

% Hint: edit controls usually have a white background on Windows.

% See ISPC and COMPUTER.

if ispc && isequal(get(hObject,'BackgroundColor'), get(0,'defaultUicontrolBackgroundColor'))

set(hObject,'BackgroundColor','white');

end

% --- Executes on button press in pushbutton1.

function pushbutton1_Callback(hObject, eventdata, handles)

% hObject handle to pushbutton1 (see GCBO)

% eventdata reserved - to be defined in a future version of MATLAB

% handles structure with handles and user data (see GUIDATA)

% handles.figure1.Name='hhh';

% --- Executes on selection change in listbox1.

function listbox1_Callback(hObject, eventdata, handles)

% hObject handle to listbox1 (see GCBO)

% eventdata reserved - to be defined in a future version of MATLAB

% handles structure with handles and user data (see GUIDATA)

% Hints: contents = cellstr(get(hObject,'String')) returns listbox1 contents as cell array

% contents{get(hObject,'Value')} returns selected item from listbox1

% --- Executes on selection change in listbox_fuzzy.

function listbox_fuzzy_Callback(hObject, eventdata, handles)

% hObject handle to listbox_fuzzy (see GCBO)

% eventdata reserved - to be defined in a future version of MATLAB

% handles structure with handles and user data (see GUIDATA)

% Hints: contents = cellstr(get(hObject,'String')) returns listbox_fuzzy contents as cell array

% contents{get(hObject,'Value')} returns selected item from listbox_fuzzy

idx = (get(handles.listbox_fuzzy,'value') );

% --- Executes during object creation, after setting all properties.

function listbox_fuzzy_CreateFcn(hObject, eventdata, handles)

% hObject handle to listbox_fuzzy (see GCBO)

% eventdata reserved - to be defined in a future version of MATLAB

% handles empty - handles not created until after all CreateFcns called

% Hint: listbox controls usually have a white background on Windows.

% See ISPC and COMPUTER.

if ispc && isequal(get(hObject,'BackgroundColor'), get(0,'defaultUicontrolBackgroundColor'))

set(hObject,'BackgroundColor','white');

end

% --- If Enable == 'on', executes on mouse press in 5 pixel border.

% --- Otherwise, executes on mouse press in 5 pixel border or over listbox1.

function listbox1_ButtonDownFcn(hObject, eventdata, handles)

% hObject handle to listbox1 (see GCBO)

% eventdata reserved - to be defined in a future version of MATLAB

% handles structure with handles and user data (see GUIDATA)

% --- Executes on button press in plotmultiple.

function plotmultiple_Callback(hObject, eventdata, handles)

% hObject handle to plotmultiple (see GCBO)

% eventdata reserved - to be defined in a future version of MATLAB

% handles structure with handles and user data (see GUIDATA)

idx = (get(handles.listbox_fuzzy,'value') );

[x,y] = get_var_names(handles);

xname = x;

xname(find(xname=='_'))=['-'];

yname = y;

yname(find(yname=='_'))=['-'];

if isempty(x) && isempty(y)

return

end

figure(gcf)

try

if (idx==1)

evalin('base',['semilogy(',y,',',x,')']), xlabel(yname),ylabel(xname);

else

evalin('base',['semilogy(',y,',',x,')']), ylabel('AlphaCuts'),xlabel(yname);

end

catch ex

errordlg(...

ex.getReport('basic'),'Error generating semilogy plot','modal')

end

% --- Executes on button press in clea_r.

function clea_r_Callback(hObject, eventdata, handles)

% hObject handle to clea_r (see GCBO)

% eventdata reserved - to be defined in a future version of MATLAB

% handles structure with handles and user data (see GUIDATA)

evalin( 'base', 'clearvars *' )

% Hint: get(hObject,'Value') returns toggle state of clea_r
